# Elevated serotonin receptor 2A signaling restores learning and memory in a Fragile X syndrome model

**DOI:** 10.1038/s41598-025-34492-4

**Published:** 2026-01-07

**Authors:** Yuchen Du, Vanessa K. Miller, Andrew J. Mellies, Kendal Broadie

**Affiliations:** 1 Departments of Biological Sciences, Vanderbilt University and Medical Center, Nashville, TN 37235 USA; 2 Cell and Developmental Biology, Vanderbilt University and Medical Center, Nashville, TN 37235 USA; 3 Pharmacology, Vanderbilt University and Medical Center, Nashville, TN 37235 USA; 4 Kennedy Center for Research on Human Development, Vanderbilt University and Medical Center, Nashville, TN 37235 USA; 5 Vanderbilt Brain Institute, Vanderbilt University and Medical Center, Nashville, TN 37235 USA

**Keywords:** Tryptophan hydroxylase, 5-hydroxytryptamine, Serotonin reuptake transporter, 5-HT_2A_ receptor, Mushroom body, *Drosophila*

## Abstract

Serotonin (5-hydroxytryptamine, 5-HT) has central roles enabling learning and memory, particularly via serotonin receptor 2A (5-HT_2A_R) signaling. *Drosophila* Fragile X syndrome model (*dfmr1* null mutant) studies reveal impaired learning and memory, which may reflect serotonergic signaling deficits. Here, we use classical olfactory T-maze conditioning to assess behavior, combined with imaging to assess 5-HT and 5-HT_2A_R levels within the underlying Mushroom Body (MB) brain circuitry. Null *dfmr1* mutants exhibit learning and memory deficits that are corrected by elevating 5-HT signaling via 1) overexpression of the serotonin biosynthetic enzyme tryptophan hydroxylase (Trhn) or 2) knockdown of the serotonin reuptake transporter (SERT). Direct comparisons reveal both Trhn and SERT manipulations equally restore learning and memory in *dfmr1* null mutants. 5-HT_2A_R levels in the MB circuit are reduced relative to controls in *dfmr1* mutants, and 5-HT_2A_R RNAi phenocopies *dfmr1* null behavioral deficits, suggesting these phenotypes are primarily caused by the loss of 5-HT_2A_R signaling. Consistently, 5-HT_2A_R overexpression in *dfmr1* nulls restores normal learning and memory compared to controls. These findings suggest loss of 5-HT_2A_R signaling causes learning and memory deficits in this Fragile X syndrome model, and that rectifying this signaling impairment can restore learning and memory, providing a framework for serotonergic intervention strategies.

**Supplementary Information:**

The online version contains supplementary material available at 10.1038/s41598-025-34492-4.

## Introduction

Fragile X syndrome (FXS) is the leading monogenetic intellectual disability (ID)^1,2^, caused predominantly by epigenetic loss of the Fragile X Messenger Ribonucleoprotein (FMRP)^3,4^. Like human FXS patients, the well-established *Drosophila* FXS disease model^5,6^ displays impairments in learning and memory^7,8^, with defects in underlying brain Mushroom Body (MB) learning/memory circuitry^9,10^ and dysfunction in multiple characterized molecular mechanisms^11–13^. This multifaceted FXS disease state may reflect truly independent FMRP functions, or could be interconnected via common foundational signaling nodes^14^. One intriguing possibility is that fundamental neuromodulator systems might compensate for impaired FXS learning/memory function^15^. Systematic brain proteomic analyses employing the *Drosophila* FXS model identified altered monoamine signaling pathways^16^ utilizing serotonin (5-hydroxytryptamine,5-HT^17,18^. We hypothesized transgenic manipulation of serotonergic signaling could prove an effective compensatory method for the treatment of learning and memory behavioral impairments in the Fragile X syndrome disease condition.

Serotonin/5-HT is a key regulator of mammalian learning and memory^19–21^. Likewise, *Drosophila* serotonergic signaling regulates learning and memory, including courtship and aversive olfactory conditioning behaviors^22,23^. The MB circuit underlying olfactory learning and memory^24,25^ receives serotonergic input and expresses conserved components of the serotonergic pathway, including 5-HT biosynthetic enzyme tryptophan hydroxylase (Trhn) and the serotonin transporter (SERT)^26–29^. Importantly, Trhn produces serotonin in neurons and glia in *Drosophila*^30^. Serotonin levels are upregulated in both FXS patients and disease models^16,18^, although this is likely compensatory as selective serotonin reuptake inhibitor (SSRI) use improves FXS symptoms^31,32^. The serotonergic 5-HT_2A_ receptor (5-HT_2A_R) helps mediate mammalian learning and memory^21,33^. *Drosophila* 5-HT_2A_Rs in neurons and glia function in olfactory circuitry^34^, but olfactory learning/memory roles have not been tested. Nothing is known about 5-HT_2A_R signaling in the *Drosophila* FXS model^35^, although 5-HT_2A_R signaling is altered in the mouse FXS model^36^, with 5-HT_2A_R proposed as a potential therapeutic target^17,18^. We hypothesized 5-HT_2A_R signaling regulates normal learning and memory, and may be impaired in the FXS condition.

Here, we employ the *Drosophila* FXS model (*dfmr1* null mutants) to systematically investigate the contributions of serotonergic signaling to learning and memory, using classic aversive olfactory conditioning coupled to MB imaging of 5-HT and 5-HT_2A_Rs. We designed experiments to probe serotonin signaling at three levels: serotonin synthesis (Trhn), serotonin uptake (SERT), and serotonin receptor (5-HT_2A_R) involvement. First, we manipulated serotonin/5-HT synthesis and reuptake using two cell-targeted transgenic methods: Trhn biosynthetic enzyme overexpression (OE) and SERT knockdown (RNAi). These complementary approaches test whether elevating serotonin alters learning and memory, and could compensate for *dfmr1* null mutant deficits. Second, 5-HT_2A_ receptors were similarly manipulated with cell-targeted 5-HT_2A_R OE and RNAi. By manipulating the 5-HT_2A_R levels, we aimed to determine whether 5-HT_2A_ receptor signaling is necessary and sufficient for normal learning/memory behavior, or could improve outcomes in the FXS model. We used classical olfactory conditioning to evaluate the effects of these serotonergic manipulations on associative learning and memory, paired to MB confocal imaging to quantify 5-HT and 5-HT_2A_R levels. This combinatory approach allows us to link neuromodulatory ligand-receptor circuit signaling to cognitive behavioral output. Our goal was to determine whether targeted serotonergic interventions could restore learning and memory in the *Drosophila* FXS genetic disease model and to determine whether 5-HT_2A_ receptor signaling contributes to learning and memory rescue in this FXS model.

## Methods

### Drosophila genetics

All stocks fed standard *Drosophila* food were reared at 25 °C in humidified incubators on a 12:12-h light/dark cycle. Adults of both sexes staged to 7–9 days post-eclosion (dpe) were used in all studies. All stocks were backcrossed to the *w*^*1118*^ background, which was used as the genetic background control. For the Fragile X syndrome (FXS) disease model, the *dfmr1*^*Δ50M*^ excision deletion null allele^6,37^ was used in combination with multiple Gal4 drivers and UAS responder lines. The Gal4 driver lines were: ubiquitous *daughterless UH1-*Gal4 (RRID: BDSC 55850)^38^, neuron-specific *elav-*Gal4 (RRID: BDSC 8765)^39^, and glia-specific *repo-*Gal4 (RRID: BDSC 7415)^40^. The UAS responder lines were: a wildtype overexpression (OE) UAS*-Trhn* line (RRID: BDSC 27638)^41^, UAS-*SERT* RNAi (RRID: VDRC 11346)^42^, UAS-*5HT*_*2A*_*R*^*OE*^ (RRID: BDSC 4830), and UAS-*5HT*_*2A*_*R* RNAi (RRID: VDRC 31882)^43^. Transgenic combinations were generated using standard *Drosophila* genetic techniques. The control lines used were: (1) genetic background *w*^*1118*^ (RRID: BDSC 3605), (2) *w*^*1118*^*,Trhn*^*OE*^*/* + *; UH1*-Gal4*/* + *,* (3) *w*^*1118*^*; SERT* RNAi*/*+*; UH1*-Gal4*/*+, (4) *w*^*1118*^*; 5HT*_*2A*_*R* RNAi*/*+*; UH1*-Gal4*/*+, and (5) *w*^*1118*^*; dfmr1*^*Δ50M*^*; UH1*-Gal4*/* + . Experimental and control studies were always conducted in parallel for all behavioral and imaging analyses.

### Behavioral analyses

**Fig. 1:**
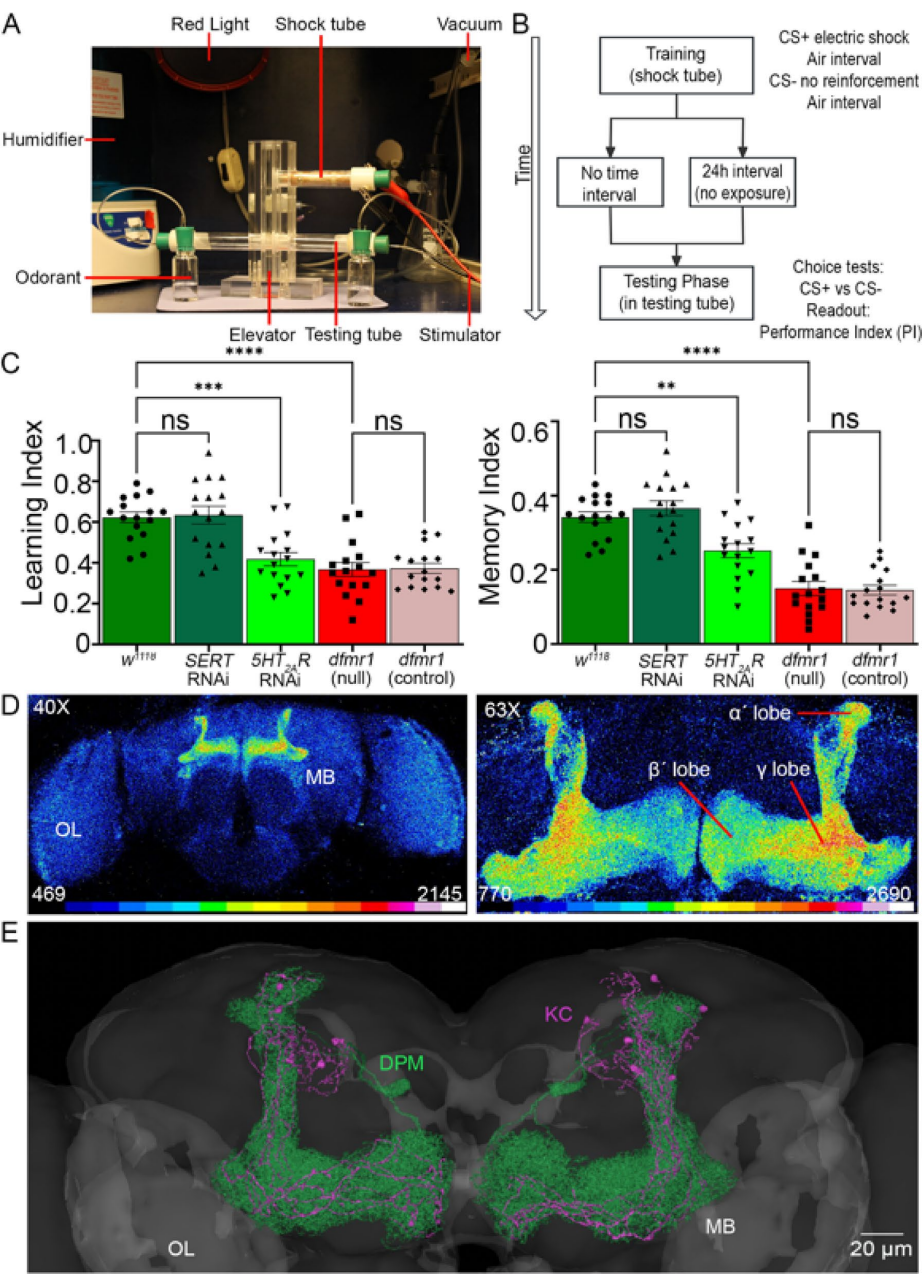
Learning and memory regulation via Mushroom Body serotonin signaling. (**A**) Aversive olfactory conditioning apparatus used for Pavlovian learning/memory assays. The setup includes red light illumination, humidity control, vacuum airflow odorant delivery, copper-grid shock tube with electrical stimulator (training), a vertical elevator for animal delivery, and a T-maze with two choice tubes (testing). Animals are trained with odorant cues paired to electric shock in the shock tube, and then scored in the T-maze test tubes. (**B**) Schematic flowchart of the learning and memory assays. The conditioning consists of alternating exposures to CS + odor paired with 12 shocks (80 V, 1.5 s duration, 5 s interval), air interval, and then the CS- odor without shock, followed by either immediate or delayed (24 h) T-maze testing. Across independent trials, OCT and MCH were assigned as CS⁺ and CS⁻ in alternative experiments. Performance index (PI = (CS^-^ – CS^+^)/(CS^-^ + CS^+^)) reports learning/memory abilities. (**C**) Quantification of learning (left) and memory (right) PIs in 5 genotypes shown: genetic background control (*w*^*1118*^); global *UH1*-Gal4 serotonin transporter (SERT) and serotonin receptor 2A (5HT_2A_R) RNAi; FXS disease model (*dfmr1* null mutant) alone and with *UH1*-Gal4/ + (*dfmr1* control). Data show individual trials (n = 16/genotype), mean ± SEM, and one-way ANOVA with Tukey’s multiple comparisons tests. For learning (left), there is a significant effect of genotype (F(4,75) = 16.54), with no significant difference between *w*^*1118*^ and *SERT* RNAi (*p* = 0.9999), while *5HT*_*2A*_*R* RNAi (*p* = 2.588 × 10^–4^) and *dfmr1* null mutants (*p* = 1.924 × 10^–6^) show reduced performance, with the *dfmr1* transgenic control not significantly different (*p* = 0.9999). For memory, there is a significant effect of genotype (F(4, 75) = 36.21), with no significant difference between *w*^*1118*^ and *SERT* RNAi (*p* = 0.9759), whereas *5HT*_*2A*_*R* RNAi (*p* = 6.347 × 10^–3^) and *dfmr1* null mutants (*p* = 3.0 × 10^–11^) show decreased performance. Again, *dfmr1* null and *dfmr1* control are not significantly different (*p* = 0.9999). The significance is indicated as *p* > 0.05 (not significant; ns), *p* < 0.01 (**), *p* < 0.001 (***), and *p* < 0.0001 (****). (**D**) *Drosophila* brain confocal images labeled with anti-Trio to reveal the Mushroom Body. Left: Whole-brain at 40 × magnification shows Mushroom Body (MB) and optic lobes (OL). Right: Higher magnification at 63 × showing MB α′, β′, and γ lobes. Fluorescence intensity is shown on a 16-color LUT scale. (**E**) Serotonergic innervation of the MB in an anatomical reconstruction from FlyWire codex. Dorsal paired medial neurons (DPM, green) provide serotonergic input onto MB Kenyon cells (KC, magenta; small subset shown for clarity).

Aversive olfactory conditioning was used to assess associative learning and memory, as previously described^8,44^. Briefly,In every trial, ~ 100 adults (7–9 days dpe) were tested for odorant conditioning behavior using a T-maze training and testing apparatus (Fig. 1A). All behavioral studies were performed in mixed sex cohorts, with the number of animals per replicate reported in Supplementary Table 1. 16 independent biological replicates were tested for each genotype and condition, with ~ 100 combined males and females per replicate (Table S1). The odorant conditioned stimulus (CS^+^) was paired to electric shock in a copper grid training tube, followed by exposure to the unconditioned odorant (CS⁻; Fig. 1B). Odorant solutions (9 μL in 8 mL mineral oil) were prepared fresh for each trial, balanced for equal aversiveness. In each individual training phase (12 trains of shock-training), a 60 s exposure to the CS⁺ (alternately 3-octanol [OCT] or 4-methylcyclohexanol [MCH]) was paired with electrical shock (1.5-s 80 V trains at 5-s intervals), then a 60 s air rest period, followed by another 60 s exposure to the CS⁻ odorant (opposing OCT or MCH) without electrical shock^44^. Immediately following training, animals were transferred within the T-maze using a central elevator (Fig. 1A,B) to test immediate odorant-shock association (defined as “learning”). Following memory training, animals were returned to food vials for a 24-h rest period and then re-introduced into the same T-maze (Fig. 1A,B) to test maintained odorant-shock association (defined as “memory”). For both trials, a 2 min period allowed choice between tubes containing CS⁺ and CS⁻ odorants. Tubes were closed, animals anesthetized using CO_2_, and then performance index (PI) was calculated blind to all genotypes using the formula: PI = CS^-^ tube number—CS^+^ tube number/total animal number. The T-maze direction and odorants (OCT or MCH) used as CS^+^ versus CS^-^ were reversed between every experiment to control for any bias.

### Immunocytochemistry imaging

Mushroom Body (MB) antibody labeling and confocal imaging was done as previously described^5^. Briefly,Adults (7–9 dpe) were anesthetized in 70% ethanol for 1 min and then brains were dissected using sharpened forceps (Dumont #5) in 1 × phosphate-buffered saline (PBS; Invitrogen). Brains were fixed for 30 min at room temperature (RT) in 4% paraformaldehyde (PFA; EMS 15714) + 4% sucrose in PBS. The fixed brains were washed 3 × with PBS and then blocked for 1.5 h at RT with 1% bovine serum albumin (BSA; Sigma-Aldrich) + 0.2% Triton X-100 in PBS (PBS-T; Fisher Chemical). Brains were then incubated with primary antibodies + 0.2% BSA in PBS-T at 4 °C overnight. Primary antibodies used: rabbit anti-5-HT (Immunostar, 20,080 1:1,000), rabbit anti-5HT_2A_R (Abcam, ab140524, 1:100), and mouse anti-Trio (Developmental Studies Hybridoma Bank (DSHB), 9.4A, 1:100). Our previous studies demonstrate anti-5HT_2A_R antibody specificity with cell-targeted *5HT*_*2A*_*R* knockdown and overexpression^30^. Brains were washed 3 × for 20 min each with PBS-T and then incubated overnight with fluorescently-conjugated secondary antibodies. The secondary antibodies used were: AlexaFluor-488 goat anti-mouse (Invitrogen, A21202, 1:250) and AlexaFluor-555 donkey anti-rabbit (Invitrogen, A31572, 1:250). Brains were washed in PBS-T 3 × for 20 min each, followed by PBS and dH_2_O 1 × for 20 min each. Brains were mounted in Fluoromount-G (EMS 17984-25) onto glass slides (75 × 25 mm, 0.9 to 1.06 mm; Corning) with a glass coverslip (No. 1.5H, Carl Zeiss). Double-sided adhesive tape (Scotch) was used to raise coverslips over the brains, with clear nail polish (Sally Hansen) used to seal the coverslip edges. All images were collected on a 510 META laser-scanning confocal microscope (Carl Zeiss) using either 40 × or 63 × oil-immersion objectives. All images were collected at 1024 × 1024 resolution with a Z-slice thickness of 0.75 μm. All confocal microscope imaging settings were kept absolutely constant in every experiment. All fluorescent measurement intensities were linear within the confocal range assayed. All the imaging quantifications were conducted blind to genotype and conditions, with the imaging analyses performed using the ImageJ Blind Analysis Tool.

### Image quantification

Confocal image measurements were performed on Mushroom Body lobes as previously described^5^. Briefly,The MB lobes were labeled with anti-Trio (DSHB 9.4A) immunocytochemistry and then the MB was manually outlined to delineate the region of interest (ROI). Fluorescence intensity quantification was conducted within the outlined MB ROI for either anti-5-HT or 5-HT_2A_R labeling as indicated, with the mean pixel fluorescence intensity values measured using NIH ImageJ as previously described^30,34^. All quantification measurements were done blind to both the genotype and training conditions using the ImageJ Blind Analysis Tool plug-in. All image acquisition settings, including the laser pinhole, gain, and offset, were held constant within every experiment. All raw images are available in the Harvard Dataverse under the "Kendal Broadie Dataverse” heading.

### Statistical analyses

**Fig. 2:**
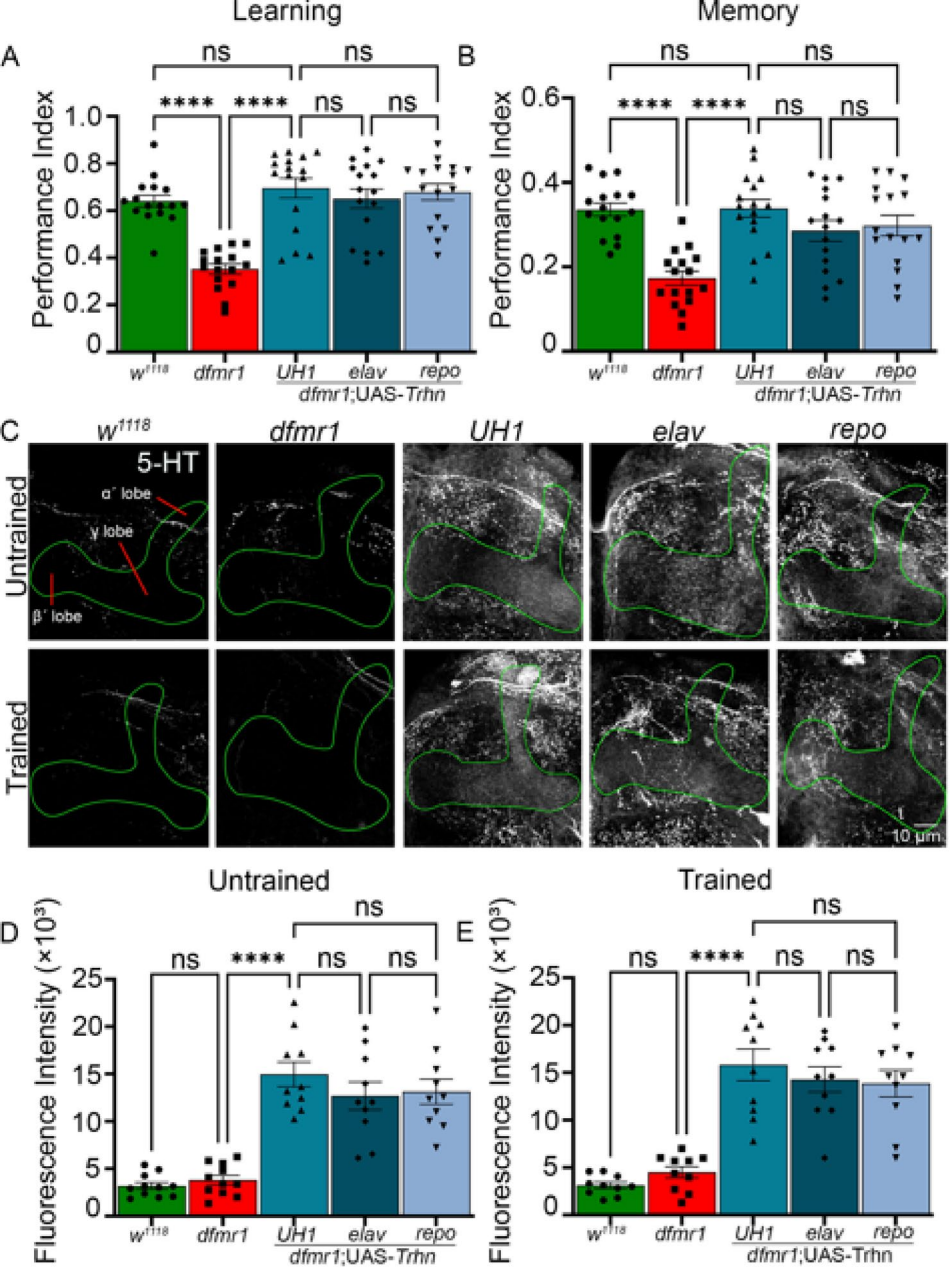
Tryptophan hydroxylase overexpression rectifies FXS learning/memory. Performance index quantification of learning (**A**) and memory (**B**) across five genotypes; genetic background control (*w*^*1118*^; green), FXS model (*dfmr1*; red), and UAS-*tryptophan hydroxylase* (*Trhn*) overexpression in the *dfmr1* background driven by *UH1-* (ubiquitous), *elav-* (neuronal), and *repo-* (glial) Gal4 lines (blue). Data show individual trials (n = 16 each), mean ± SEM, and one-way ANOVA with Tukey’s multiple comparisons tests. Learning performance shows a significant effect of genotype (F(4,75) = 17.93). For learning, a significant impairment occurs between *w*^*1118*^ and *dfmr1* (*p* = 4.662 × 10^–7^), and a significant improvement between *dfmr1* alone and with *UH1-*Gal4 driven UAS-*Trhn* (*p* = 3.367 × 10^–9^). No significant differences occur in any other comparisons. Memory performance also shows a significant effect of genotype (F(4,75) = 10.58). For memory trials, a significant impairment occurs between *w*^*1118*^ and *dfmr1* (*p* = 3.902 × 10^–6^), and significant improvement between *dfmr1* alone and with *UH1-*Gal4 driven UAS-*Trhn* (*p* = 2.645 × 10^–6^). No significant differences occur in any other comparisons. (**C**) Representative Mushroom Body lobe (anti-Trio, green outline) anti-serotonin (5-HT, grey scale) labeling in the same five above genotypes in untrained (top row) and trained (bottom row) conditions. Quantification of MB 5-HT fluorescence intensity from untrained (**D**) and T-maze trained (**E**) conditions. Data show individual brains (n = 10–15/condition), mean ± SEM, and one-way ANOVA with Tukey’s multiple comparisons tests. For the untrained basal condition, the overall effect of genotype is significant (F(4, 47) = 28.50), with a significant elevation occurring between *dfmr1* nulls alone versus with *UH1-*Gal4 driven UAS-*Trhn* (*p* = 1.477 × 10^–8^). No significant differences occur in any other comparisons. For the T-maze trained condition, the overall effect of genotype is also significant (F(4, 45) = 25.75), with a significant increase between *dfmr1* nulls alone versus with *UH1-*Gal4 driven UAS-*Trhn* (*p* = 2.207 × 10^–7^). No significant differences occur in any other comparisons. Significance is indicated as *p* > 0.05 (not significant; ns) and *p* < 0.0001 (****).

**Fig. 3:**
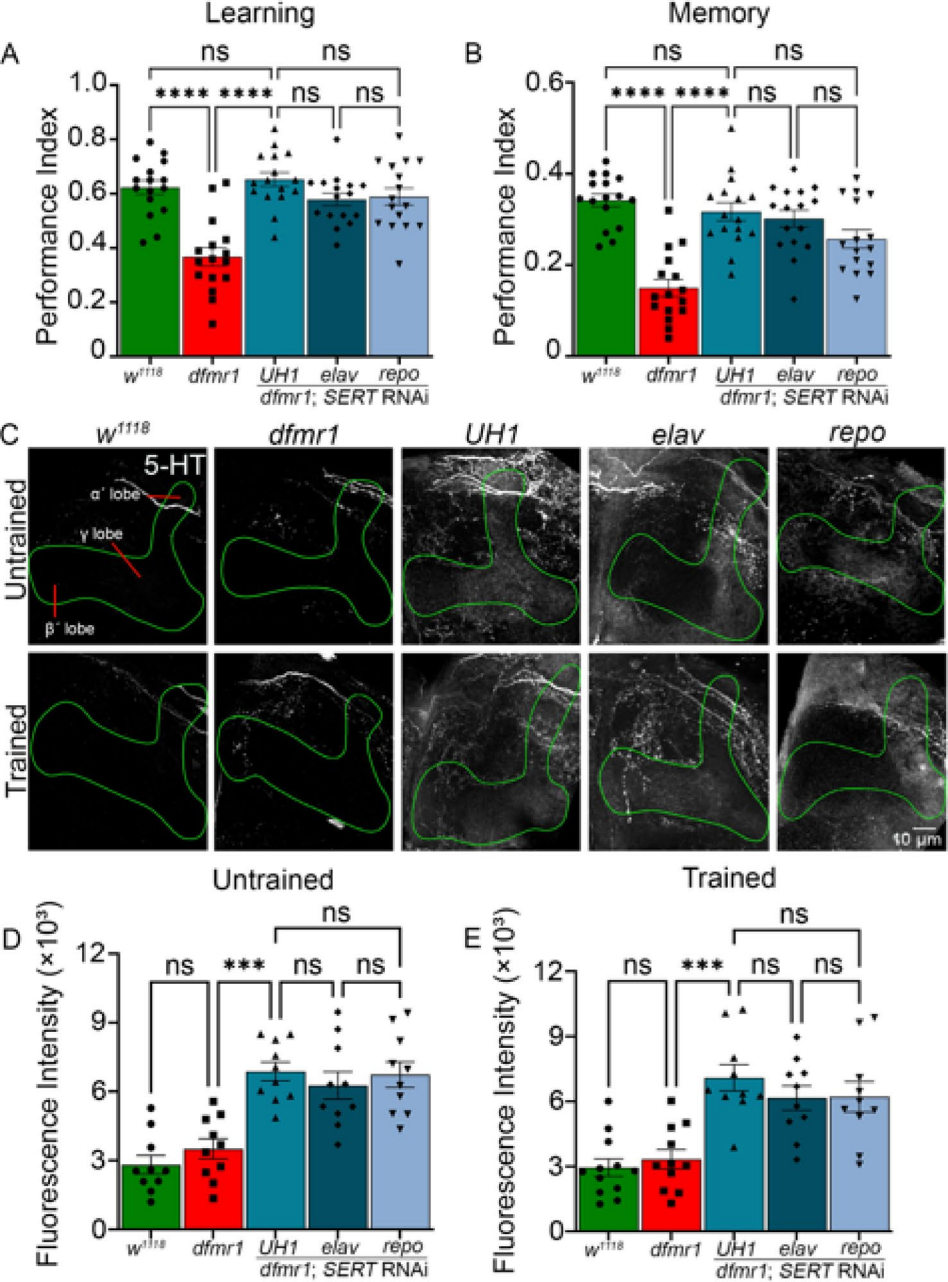
Serotonin transporter (SERT) knockdown restores FXS learning/memory. Learning (**A**) and memory (**B**) performance index quantifications across five genotypes; genetic background control (*w*^*1118*^; green), FXS model (*dfmr1*; red), and UAS-*serotonin transporter* (*SERT*) RNAi in the *dfmr1* mutant background driven by *UH1-* (ubiquitous), *elav-* (neuronal), and *repo-* (glial) Gal4 lines (blue). Data show individual trials (n = 16 each), mean ± SEM, and one-way ANOVA with Tukey’s multiple comparisons tests. Learning performance shows a significant effect of genotype (F(4,75) = 16.40). For learning, a significant impairment occurs between *w*^*1118*^ and *dfmr1* (*p* = 8.993 × 10^–6^), and a significant improvement between *dfmr1* alone vs with *UH1-*Gal4 driven *SERT* RNAi (*p* = 4.388 × 10^–7^). No significant differences occur in any of the other comparisons. Memory performance also shows a significant effect of genotype (F(4,75) = 17.00). For memory, a significant impairment occurs between *w*^*1118*^ and *dfmr1* (*p* = 6.184 × 10^–9^), and significant improvement between *dfmr1* alone and with *UH1-*Gal4 driven UAS-*SERT* RNAi (*p* = 6.644 × 10^–7^). No significant differences occur in any other comparisons. (**C**) Mushroom Body lobe outline (anti-Trio, green) and anti-serotonin (5-HT, grey scale) labeling in the same five genotypes in untrained (top row) and trained (bottom row) conditions. Quantification of the MB 5-HT fluorescence intensity in both the untrained (**D**) and trained (**E**) conditions. Data show individual brains (n = 10–15/condition), mean ± SEM, and one-way ANOVA with Tukey’s multiple comparisons tests. For the untrained condition, the overall effect of genotype is significant (F(4, 45) = 15.62), with a significant elevation occurs between *dfmr1* alone and with *UH1-*Gal4 driven UAS-*SERT* RNAi (*p* = 1.285 × 10^–4^). No significant differences occur in any of the other comparisons. For the trained condition, the overall effect of genotype is also significant (F(4, 48) = 12.19), with a significant increase likewise occurring between *dfmr1* alone and with *UH1-*Gal4 driven UAS-*SERT* RNAi (*p* = 1.267 × 10^–4^). No significant differences occur in any other comparisons. Significance is indicated as *p* > 0.05 (not significant; ns), *p* < 0.001 (***), and *p* < 0.0001 (****).

**Fig. 4:**
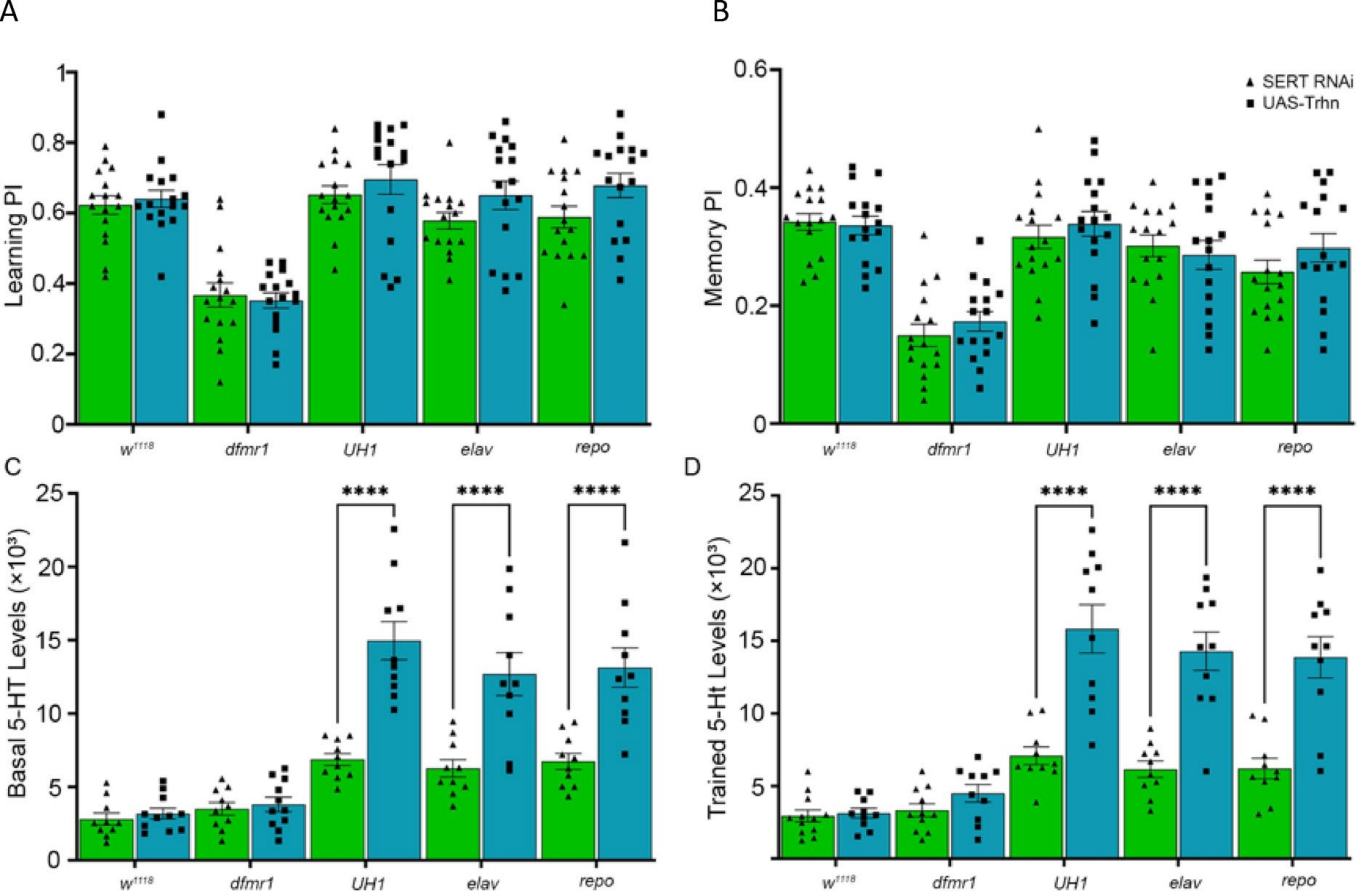
Comparison of the *Trhn* overexpression and *SERT* knockdown effects. Quantification of performance index in learning (**A**) and memory (**B**) assays by genotype, tested with either *Trhn* overexpression (OE, blue) or *SERT* knockdown (RNAi, green). Genotypes include genetic background control (*w*^*1118*^), FXS model (*dfmr1*), and both UAS transgenes in the *dfmr1* null background driven by *UH1-* (ubiquitous), *elav-* (neuronal), and *repo-* (glial) Gal4 lines. Data show individual trials (n = 16 per condition), mean ± SEM, and one-way ANOVA with Tukey’s multiple comparisons tests. For learning, the two-way ANOVA reveals no significant interaction between genotype and training (F(4,150) = 0.9138), a significant effect of genotype (F(4,150) = 33.16), and no significant effect of *Trhn* overexpression versus *SERT* knockdown (F(1,150) = 4.454). Similarly, for memory, the two-way ANOVA reveals no significant interaction between genotype and training (F(4,150) = 0.7009), a significant effect of genotype (F(4,150) = 26.12), and no significant effect of Trhn overexpression versus SERT knockdown (F(1,150) = 1.093). Quantification of basal (**C**) and trained (**D**) 5-HT fluorescence intensities in the Mushroom Body across the same genotypes and conditions. Data points show results from individual brains (n = 10–15 per condition), mean ± SEM, and one-way ANOVA with Tukey’s multiple comparisons tests. For the basal condition, the two-way ANOVA shows no significant interaction between genotype and training (F(4,92) = 9.901), a significant main effect of genotype (F(4,92) = 40.85), and no significant main effect of *Trhn* overexpression versus *SERT* RNAi (F(1,92) = 66.85). In the basal condition, significant differences in 5-HT intensity occur between *Trhn* OE and *SERT* RNAi with *UH1-* (*p* = 4.785 × 10^–8^), *elav-* (*p* = 2.325 × 10^–5^), and *repo-* (*p* = 2.528 × 10^–5^) Gal4 drivers. For the trained condition, the two-way ANOVA likewise shows no significant interaction (F(4,93) = 10.64), significant effect of genotype (F(4,93) = 38.50), and no significant effect of training (F(1,93) = 81.86). In the trained condition, significant differences also occur between *Trhn* OE and *SERT* RNAi with *UH1-* (*p* = 5.731 × 10^–8^), *elav-* (*p* = 4.934 × 10^–7^), and *repo-* (*p* = 2.470 × 10^–6^) drivers. Significance is indicated as p < 0.0001 (****).

**Fig. 5:**
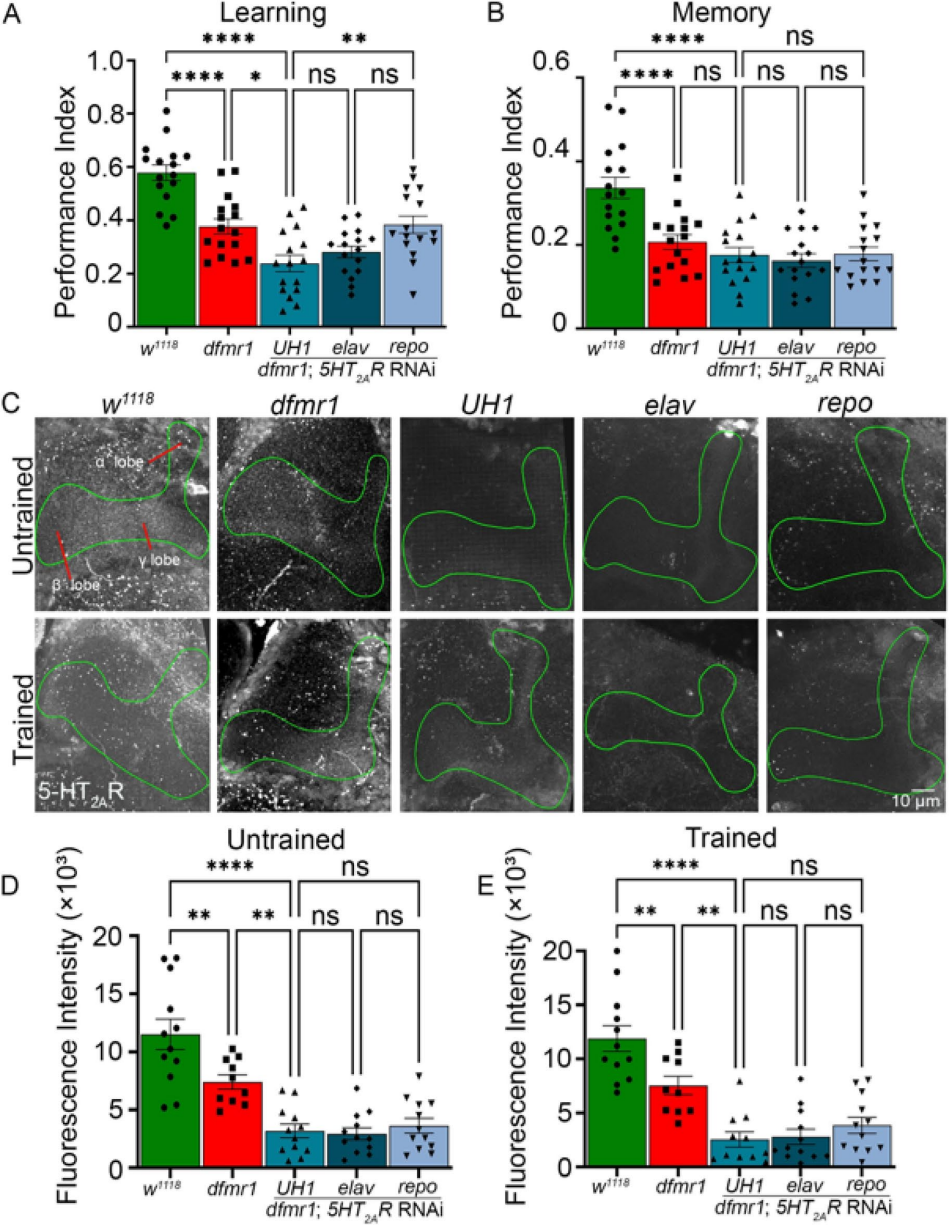
*5HT*_*2A*_*R* knockdown only weakly exacerbates FXS model learning/memory. Performance index quantifications of learning (**A**) and memory (**B**) across five genotypes; the genetic background control (*w*^*1118*^; green), FXS model (*dfmr1*; red), and UAS-*5HT*_*2A*_* receptor* (*5HT*_*2A*_*R*) RNAi in the *dfmr1* null background driven by *UH1-* (ubiquitous), *elav-* (neuronal), and *repo-* (glial) Gal4 lines (blue). Data show all individual trials (n = 16 each), mean ± SEM, and one-way ANOVA with Tukey’s multiple comparisons tests. Learning performance shows a significant effect of genotype (F(4,75) = 21.02). For learning, significant differences occur between *w*^*1118*^ and *dfmr1* (*p* = 7.460 × 10^–5^), *w*^*1118*^ and *UH1*-Gal4 driven *5HT*_*2A*_*R* RNAi in the *dfmr1* null mutant (*p* = 4.617 × 10^–10^), and between this condition and *repo*-Gal4 driven *5HT*_*2A*_*R* RNAi in the *dfmr1* mutant (*p* = 9.559 × 10^–3^). A significant difference also occurs between *dfmr1* and *UH1*-Gal4 driven *5HT*_*2A*_*R* RNAi in the *dfmr1* mutant (*p* = 0.01548). No significant differences occur in the other comparisons. Memory performance also shows a significant effect of genotype (F(4,75) = 14.04). For memory, significant differences occur between *w*^*1118*^ and *dfmr1* (*p* = 8.599 × 10^–5^), and between *w*^*1118*^ and *UH1*-Gal4 driven *5HT*_*2A*_*R* RNAi in the *dfmr1* mutant (*p* = 7.094 × 10^–7^). No other significant differences occur. (**C**) Representative Mushroom Body lobe (anti-Trio, green outline) anti-serotonin receptor 2A (5HT_2A_R, grey scale) labeling in the same five genotypes in untrained (top row) and trained (bottom row) conditions. Quantification of MB 5-HT fluorescence intensity from untrained (**D**) and trained (**E**) conditions. Data show individual brains (n = 10–15 per condition), mean ± SEM, and one-way ANOVA with Tukey’s multiple comparisons tests. For the basal untrained condition, the overall effect of genotype is significant (F(4, 53) = 21.92), with a significant difference occurs between *w*^*1118*^ and *dfmr1* (*p* = 7.858 × 10^–3^), *w*^*1118*^ and *UH1*-Gal4 driven *5HT*_*2A*_*R* RNAi in the *dfmr1* mutant (*p* = 7.621 × 10^–9^), and *dfmr1* and *UH1*-Gal4 driven *5HT*_*2A*_*R* RNAi in the *dfmr1* mutant (*p* = 5.732 × 10^–3^). No significant differences occur in the other comparisons. For the trained condition, the overall effect of genotype is significant (F(4, 52) = 21.58), with a significant 5HT_2A_R loss occurring between *w*^*1118*^ and *dfmr1* null (*p* = 9.630 × 10^–3^), *w*^*1118*^ and *UH1*-Gal4 driven *5HT*_*2A*_*R* RNAi in the *dfmr1* null mutant (*p* = 5.083 × 10^–9^), and *dfmr1* and *UH1*-Gal4 driven *5HT*_*2A*_*R* RNAi in the *dfmr1* null mutant (*p* = 2.454 × 10^–3^). No other significant differences occur. Significance is indicated as *p* > 0.05 (not significant, ns), *p* < 0.05 (*), *p* < 0.01 (**), and *p* < 0.0001 (****).

**Fig. 6:**
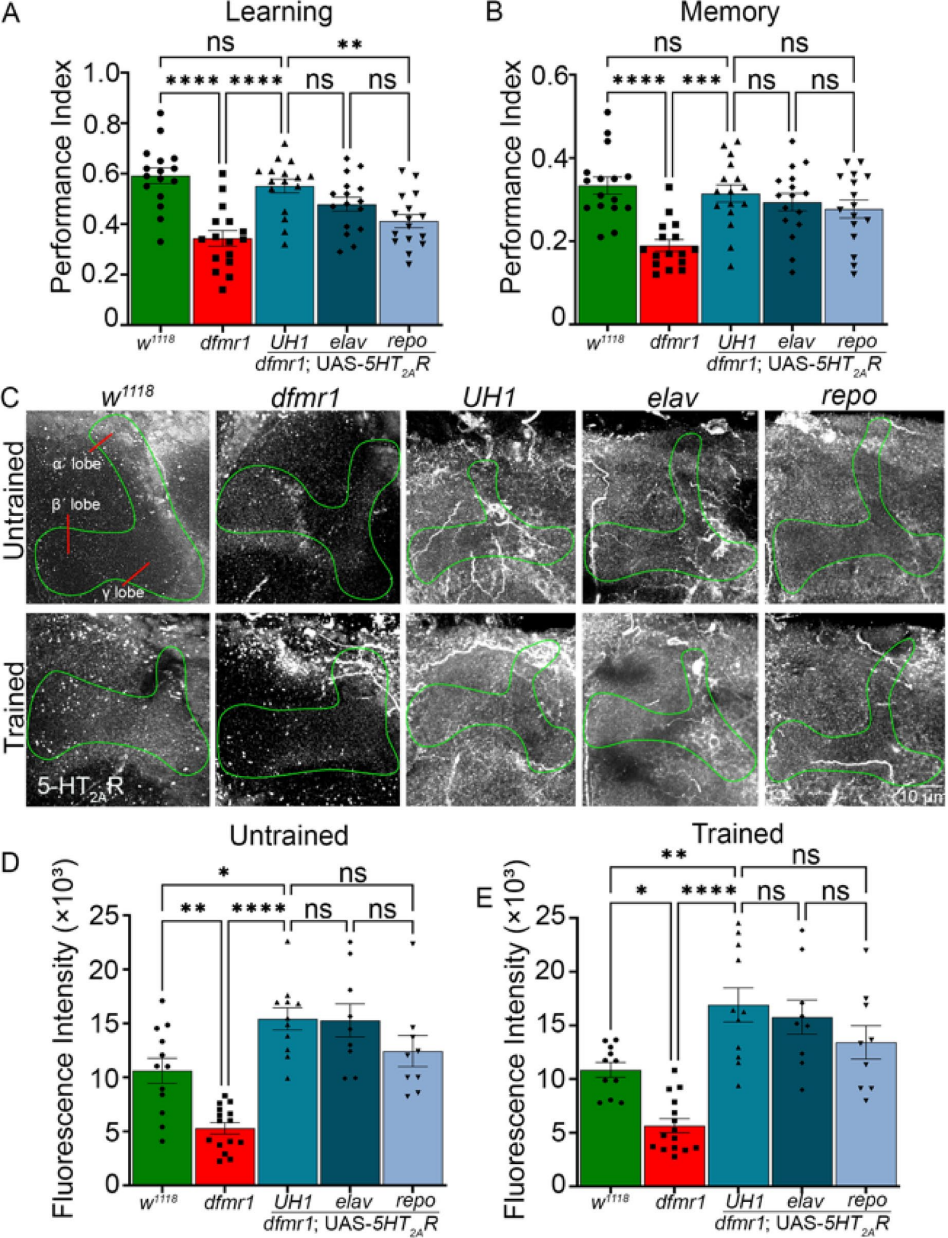
*5HT*_*2A*_*R* overexpression rectifies FXS model learning/memory performance. Learning (**A**) and memory (**B**) performance index quantifications across five genotypes; genetic background control (*w*^*1118*^; green), FXS model (*dfmr1*; red), and UAS-*5HT*_*2A*_* receptor* (*5HT*_*2A*_*R*) overexpression in the *dfmr1* background driven by *UH1-* (ubiquitous), *elav-* (neuronal), and *repo-* (glial) Gal4 lines (blue). Data show individual trials (n = 16 each), mean ± SEM, and one-way ANOVA with Tukey’s multiple comparisons tests. Learning performance shows a significant effect of genotype (F(4,75) = 21.30). For learning, a significant impairment occurs between *w*^*1118*^ and *dfmr1* (*p* = 4.112 × 10^–7^), improvement compared to *dfmr1* with *UH1*-Gal4 driven UAS-*5HT*_*2A*_*R* in the *dfmr1* null (*p* = 2.404 × 10^–5^), and decrease from the *UH1-* to *repo*-Gal4 conditions (*p* = 8.799 × 10^–3^). Memory performance also shows a significant effect of genotype (F(4,75) = 7.881). For memory, a significant loss again occurs between *w*^*1118*^ and *dfmr1* (*p* = 2.133 × 10^–5^), which is restored comparing *dfmr1* to *UH1*-Gal4 driven UAS-*5HT*_*2A*_*R* in the *dfmr1* null (*p* = 2.762 × 10^–4^). No significant differences occur in the other comparisons. (**C**) Mushroom Body lobe (anti-Trio, green outline) anti-serotonin receptor 2A (5HT_2A_R, grey scale) labeling in the same five genotypes in the untrained (top row) and trained (bottom row) conditions. Quantification of the MB 5-HT_2A_R fluorescence intensity in the untrained (**D**) and trained (**E**) conditions. Data show individual brains (n = 10–15 per condition), mean ± SEM, and one-way ANOVA with Tukey’s multiple comparisons tests. For the untrained condition, the overall effect of genotype is significant (F(4, 51) = 16.85), with a significant decrease occurs between *w*^*1118*^ and *dfmr1* (*p* = 3.495 × 10^–3^), *w*^*1118*^ and *UH1*-Gal4 driven UAS-*5HT*_*2A*_*R* in *dfmr1* (*p* = 2.067 × 10^–2^), and between *dfmr1* and *UH1*-Gal4 driven UAS-*5HT*_*2A*_*R* in *dfmr1* (*p* = 4.722 × 10^–8^). For the trained condition, the overall effect of genotype is significant (F(4, 50) = 16.48), with a significant loss between *w*^*1118*^ and *dfmr1* (*p* = 1.423 × 10^–2^), *w*^*1118*^ and *UH1*-Gal4 driven UAS-*5HT*_*2A*_*R* in *dfmr1* (*p* = 6.309 × 10^–3^), and between *dfmr1* and *UH1*-Gal4 driven UAS-*5HT*_*2A*_*R* in the *dfmr1* null (*p* = 3.127 × 10^–8^). No significant differences occur in other comparisons. Significance is indicated as *p* > 0.05 (not significant, ns), *p* < 0.05 (*), *p* < 0.01 (**), *p* < 0.001 (***), and *p* < 0.0001 (****).

All comparisons were performed using Prism software (GraphPad version 9). For the different experiments, one-way analysis of variance (ANOVA) (Figs. 1, 2, 3, 5, and 6) or two-way ANOVA (Fig. 4) were used to assess changes among genotypes or treatment groups, followed by Tukey’s multiple comparisons tests for multiple group comparisons. Sample sizes are specified in the figure legends. For behavioral assays, each data point represents one trial consisting of ~ 100 animals. For imaging assays, each data point represents one Mushroom Body lobe in a different brain. All the data meet normality and homogeneity of variance ANOVA requirements. Normality tests were conducted for every comparison. All datasets passed these criteria by using D’ Agostino & Pearson tests. Data are presented as mean ± standard error of the mean (SEM). All mean ± SEM numerical values for both behavioral and imaging datasets are provided in Supplementary Table 1, with exact *p*-values reported. Significance in figures is indicated as *p* < 0.05 (*), *p* < 0.01 (**), *p* < 0.001 (***), and *p* < 0.0001 (****). Values of *p* > 0.05 are reported as not significant (ns). The exact *p*-values for all significant comparisons are also provided in the figure legends.

## Results

### The ***Drosophila*** FXS model and ***5HT***_***2A***_***R*** knockdown share learning/memory deficits

*Drosophila* olfactory conditioning has been used for decades to dissect the genetic mechanisms of learning and memory^8,44^. Classical aversive conditioning is done in a T-maze with a conditioned stimulus (CS^+^) odor paired to electric shock training versus an unconditioned stimulus (CS^−^) odor, and then tests for the ability to discriminate between them (Fig. 1A). Testing can be done either immediately (“learning”) or after a delay (“memory”) to separate these two different behavioral phases (Fig. 1B). Performance index (PI) outcomes are quantified as (CS^–^ − CS^+^)/(CS^–^ + CS^+^) for both learning and memory (Fig. 1C). To begin to test links between serotonin signaling and the FXS disease model (*dfmr1* null mutants), 5 genotypes were tested: the genetic background control (*w*^*1118*^), a serotonin transporter knockdown (*SERT* RNAi), a serotonin receptor 2A knockdown (*5HT*_*2A*_*R* RNAi), *dfmr1* null mutants (*dfmr1*^*∆50 M*^), and transgenic *UH1*-Gal4/ + driver in this background (*dfmr1* control). Compared to genetic background controls, *UH1*-Gal4 driven *SERT* RNAi has no significant effect on either the immediate learning or maintained memory outcomes (Fig. 1C), suggesting that elevated serotonin (5-HT) levels do not impact baseline behavioral performance. In contrast, *5HT*_*2A*_*R* RNAi significantly impairs both immediate learning (*p* = 2.588 × 10^–4^) and memory maintenance (*p* = 6.347 × 10^–3^), showing that reduced *5HT*_*2A*_*R* signaling causes learning and memory deficits (Fig. 1C). Similarly, the *dfmr1* null mutants have both significantly reduced learning (*p* = 1.924 × 10^–6^) and memory (*p* = 3.0 × 10^–11^), which are unaltered with the transgenic Gal4 driver control (Fig. 1C). These findings indicate *5HT*_*2A*_*R* and *dfmr1* loss similarly impair immediate learning, and both cause strong memory deficits, suggesting a possible role of serotonergic signaling in *Drosophila* FXS disease model behavioral phenotypes.

The *Drosophila* central brain Mushroom Body (MB) is the site of learning/memory circuitry (Fig. 1D, left)^45^. The MB circuit is composed of approximately 2,000 intrinsic MB neurons/hemisphere called Kenyon cells (KCs)^46^, which extend their axons into discrete MB lobes (α/β, α′/β′, and γ; Fig. 1D, right) to form compartmentalized circuits that mediate associative learning and memory consolidation^47^. The KC γ neurons primarily mediate odorant associative learning^48^, the α/β neurons contribute to memory retrieval^49,50^, and the α′/β′neurons are essential for long-term memory (LTM) storage^50^. To study the MB circuit underlying the behavioral outcomes, staged brains were visualized with anti-Trio labeling^5,51^, which reveals the MB lobes in the context of the whole brain (Fig. 1D). High magnification imaging shows the α′, β′, and γ lobes that are the focus of our serotonin pathway studies (Fig. 1D, right). The recently released FlyWire codex allows complete 3D reconstruction to investigate MB serotonergic circuitry^52^. The serotonergic dorsal paired medial neuron (DPM, green) provides the principal MB serotonergic input (Fig. 1E. DPMs directly innervate MB Kenyon cells (magenta; only a small subset of KCs shown for clarity, providing widespread serotonergic modulation driving learning acquisition and memory consolidation^27,53^. The FlyWire connectome confirms extensive DPM processes overlapping the MB intrinsic circuitry, with defined synaptic inputs onto MB Kenyon cells, illuminating the spatial organization of serotonergic innervation in relation to this well-defined learning and memory circuit (Fig. 1E). Based on this behavioral and brain MB circuitry framework, we set forth to test the role of serotonergic signaling in *Drosophila* FXS model learning and memory impairments.

### Tryptophan hydroxylase overexpression restores FXS model learning and memory

The rate-limiting serotonin biosynthetic enzyme is tryptophan hydroxylase (Trhn)^54^. *Trhn* overexpression elevates 5-HT levels and potentiates serotonergic signaling^30,55^. To investigate whether increasing serotonin could modulate learning/memory impairments in the *Drosophila* FXS disease model (*dfmr1* null mutants), we overexpressed UAS-*Trhn*^41^ using three drivers: ubiquitious (*UH1*-Gal4), neuronal (*elav*-Gal4), and glial (*repo*-Gal4). qPCR testing *Trhn* overexpression (OE) demonstrates this transgenic method is highly efficacious (*UH1*-Gal4/*w*^*1118*^ vs. *UH1*-Gal4; UAS-*Trhn* ∆∆C_q_ 14.31 ± 0.108, *p* = 1.962 × 10^–7^). All *Trhn* overexpression genotypes were tested alongside Gal4 driver (*UH1*-Gal4/*w*^*1118*^, *elav*-Gal4/*w*^*1118*^, *repo*-Gal4/*w*^*1118*^) as well as the UAS transgene (UAS-*Trhn*/*w*^*1118*^) controls. Compared to the genetic background control (*w*^*1118*^), *dfmr1* null mutants have a significant learning impairment (*p* = 4.662 × 10^–7^), which can be restored by *UH1*-Gal4 driven *Trhn* overexpression, significantly improving learning relative to the *dfmr1* nulls (*p* = 3.367 × 10^–9^) and resulting in a performance that shows no significant difference from *w*^*1118*^ controls (*p* = 0.7789; Fig. 2A). Consistently, neuronal *elav*-Gal4 driven *Trhn* overexpression results in a comparable improvement in FXS model learning, and we were surprised that glial overexpression also proved efficacious (Fig. 2A, right). For memory compared to controls, ubiquitous *Trhn* overexpression in *dfmr1* mutants restores performance (*p* = 3.902 × 10^–6^) and improves memory over *dfmr1* nulls (*p* = 2.645 × 10^–6^), with no significant difference left to genetic background controls (*p* = 0.9999; Fig. 2B). As above, neuronal (*elav*-Gal4) and glial (*repo*-Gal4) *Trhn* overexpression in *dfmr1* mutants similarly corrects memory, with no significant differences among the three drivers, albeit with a trend towards an additive effect from neuronal *elav*-Gal4 and glial *repo*-Gal4 compared to the ubiquitous *UH1*-Gal4 (Fig. 2B, right). The 3 Gal4 driver and UAS-Trhn controls showed no behavior differences compared to the *w*^*1118*^ genetic background. These findings suggest elevated serotonin signaling is sufficient to restore learning/memory in the *Drosophila* FXS model.

To assay serotonin signaling in the underlying Mushroom Body (MB) circuitry, all genotypes were labeled with anti-5-HT^30^, both under baseline, untrained conditions and following the above training protocol (Fig. 2C). Serotonin levels were imaged by delineating the MB with anti-Trio labeling^5^. Anti-Trio images used to delineate the MB lobes (green outlines, Fig. 2C) are shown for all genotypes and conditions (Fig. S1). The genetic background control (*w*^*1118*^) and FXS disease model (*dfmr1* null mutants) exhibit low basal levels of serotonin, with *Trhn* overexpression dramatically elevating serotonin (Fig. 2C, top row). T-maze aversive olfactory conditioning training does not detectably alter serotonin in any of the genotypes (Fig. 2C, bottom row). Anti-Trio immunostaining was performed in all trials, but was not displayed for clarity; the anti-trio outline was used to define the MB quantification region. The anti-5-HT fluorescence intensity was quantified within the MB (sum of slices) to quantitatively compare serotonin levels. In untrained conditions, there is no significant difference between control and *dfmr1* nulls (*p* = 0.9927), indicating that basal serotonin levels are not detectably affected by *dfmr1* loss alone (Fig. 2D, left). Compared to the *dfmr1* mutants, the *Trhn* overexpression results in a highly significant elevation of serotonin in the MB (*UH1*-Gal4 driven *Trhn* overexpression; *p* = 1.477 × 10^–8^), with a similar elevation from the other drivers (Fig. 2D, right). In the trained condition, results are similar, with no significant difference between *w*^*1118*^ and *dfmr1* nulls (*p* = 0.9231), compared to a highly significant serotonin elevation arising from *UH1*-Gal4 driven *Trhn* overexpression (*p* = 2.207 × 10^–7^; Fig. 2E). There is a trend towards an additive effect from *elav*- and *repo*-Gal4 versus ubiquitous *UH1*-Gal4, but no significant differences (Fig. 2D,E). All 3 Gal4 (*UH1*-Gal4/*w*^*1118*^, *elav*-Gal4/*w*^*1118*^, *repo*-Gal4/*w*^*1118*^) and UAS transgene (UAS-*Trhn*/*w*^*1118*^) controls display 5-HT levels indistinguishable from the *w*^*1118*^ background control (Fig. S2). Taken together, these findings suggest elevating brain serotonin levels restores learning and memory to control levels within detectable limits in the *Drosophila* FXS disease model.

### Serotonin transporter knockdown likewise corrects FXS model learning/memory

To independently test whether increasing serotonin signaling can correct learning and memory deficits in the *dfmr1* null mutants—and to distinguish whether serotonin synthesis or reuptake contributes differentially to this restorative effect—we next tested whether inhibiting the serotonin transporter (SERT) could replicate the behavioral and molecular changes compared to *Trhn* overexpression. SERT inhibition prevents serotonin reuptake to elevate serotonin levels in the brain^56^. We employed a UAS-*SERT* RNAi^42^ in the *dfmr1* null mutants using the same three Gal4 drivers above to transgenically control serotonin reuptake in our FXS disease model. All *SERT* RNAi genotypes were tested beside both Gal4 drivers alone (*UH1*-Gal4/*w*^*1118*^, *elav*-Gal4/*w*^*1118*^, *repo*-Gal4/*w*^*1118*^) and UAS-transgene (*SERT* RNAi/*w*^*1118*^) controls, which all performed at *w*^*1118*^ levels (Fig. 1C). Compared to the genetic background control (*w*^*1118*^), the *dfmr1* null mutants again exhibit significant learning (*p* = 8.993 × 10^–6^) and memory (*p* = 6.184 × 10^–9^) impairments (Fig. 3A,B). Ubiquitous *SERT* knockdown in the *dfmr1* null mutants (*UH1*-Gal4 driven UAS-*SERT* RNAi) significantly improves learning performance compared to the *dfmr1* null mutants (*p* = 4.388 × 10^–7^), restoring behavior that is statistically indistinguishable from the genetic controls (*p* = 0.9998; Fig. 3A, middle). Likewise, global *UH1*-driven *SERT* RNAi significantly improves memory performance compared to the *dfmr1* null mutants (*p* = 6.644 × 10^–7^), correcting memory performance back to control levels, with no statistical difference remaining compared to the *w*^*1118*^ genetic background animals (*p* = 0.9960; Fig. 3B, middle). Neuronal (*elav*-Gal4) and glial (*repo*-Gal4) *SERT* knockdown show similar restoration of learning and memory, with no significant differences between any of the three different Gal4 driver lines (Fig. 3A,B). These findings suggest that serotonin transporter inhibition (*SERT* RNAi) is sufficient to restore both learning and memory performance in the *Drosophila* FXS disease model.

To test whether *SERT* knockdown increases brain serotonin levels as expected, we performed anti-5-HT labeling in the basal untrained and T-maze trained conditions in all the genotypes (Fig. 3C). All of the control genotypes (*UH1*-Gal4/*w*^*1118*^, *elav*-Gal4/*w*^*1118*^, *repo*-Gal4/*w*^*1118*^, and UAS-*SERT* RNAi/* w*^*1118*^) display 5-HT levels comparable to *w*^*1118*^, indicating that any observed changes are specific to *SERT* knockdown (Fig. S2). With MB anti-Trio labeling, serotonin levels are again very low in genetic background control (*w*^*1118*^) and FXS model (*dfmr1* nulls) in both treatment conditions (Fig. 3C). *SERT* RNAi increases serotonin levels within the MB, but to a much lower extent than the previous *Trhn* overexpression effects (compare Figs. 2C and 3C). T-maze aversive conditioning training does not detectably alter serotonin compared to baseline in any of the genotypes (Fig. 3C, bottom row). Anti-Trio immunostaining was performed in all trials, with the outline displayed used for quantification. In the anti-Trio defined MB, the anti-5-HT fluorescence intensity was quantified across untrained and trained conditions. In the basal state, there is again no significant differences between *w*^*1118*^ controls and *dfmr1* mutants (*p* = 0.8508), but serotonin levels are significantly elevated by ubiquitous *SERT* knockdown in *dfmr1* mutants (*UH1*-Gal4 *SERT* RNAi) compared to *dfmr1* alone (*p* = 1.285 × 10^–4^; Fig. 3D). There are no significant differences between the three *SERT* knockdown conditions, suggesting that the serotonin transporter operates in both neurons and glia to limit 5-HT levels in the MB. With T-maze training, there is still no significant difference in MB serotonin levels in the *w*^*1118*^ controls and *dfmr1* mutants (*p* = 0.9839), but *UH1*-driven *SERT* RNAi again significantly increases 5-HT levels compared to the *dfmr1* nulls (*p* = 1.267 × 10^–4^; Fig. 3E). There is a trend for additive effect from *elav*-Gal4 and *repo*-Gal4 towards *UH1*-Gal4, but no significant differences. Taken together, these findings suggest elevating serotonin by two independent methods restores learning/memory in the *Drosophila* FXS model.

Elevating serotonin levels via *Trhn* overexpression (increasing 5-HT synthesis) or *SERT* knockdown (reducing 5-HT reuptake) appears to produce comparable restoration of FXS model learning and memory. To weigh the relative efficacy of these two strategies, we directly compare the UAS-*Trhn* overexpression (blue) and UAS-*SERT* RNAi (green) effects on *dfmr1* mutants (Fig. 4). Quantification of the learning performance index (PI) reveals that both approaches produce comparable results, with no significant differences between the genetic background controls (*w*^*1118*^), FXS model (*dfmr1*), or the transgenes driven with any of the three Gal4 drivers (Fig. 4A). Likewise, memory performance was indistinguishable in both manipulations, with no significant differences between the five genotypes with either *Trhn* overexpression or *SERT* knockdown (Fig. 4B). These findings suggest that both transgenic strategies effectively restore learning and memory in *dfmr1* null mutants, and that neither method yields more benefit. However, 5-HT analyses reveal significant differences in MB serotonin elevation with the two approaches. In the basal, untrained condition, *Trhn* overexpression promotes significantly greater serotonin levels compared to *SERT* RNAi under *UH1-* (*p* = 4.785 × 10^–8^), *elav-* (*p* = 2.325 × 10^–5^), and *repo-* (*p* = 2.528 × 10^–5^) Gal4 driver expression (Fig. 4C). For the T-maze trained condition, *Trhn* overexpression continues to elevate serotonin more than the *SERT* RNAi counterparts between *UH1-* (*p* = 5.731 × 10^–8^), *elav-* (*p* = 4.934 × 10^–7^), and *repo-* (*p* = 2.470 × 10^–6^) drivers (Fig. 4D). These findings show *Trhn* overexpression and *SERT* knockdown both restore behavioral performance, but with distinct magnitudes of serotonin elevation. We next turned to testing the role of serotonin receptors within our FXS disease model.

### Serotonin receptor 2A knockdown phenocopies FXS model learning and memory

The behavioral correction of *dfmr1* mutants with *Trhn* overexpression or *SERT* knockdown suggests increasing serotonin signaling can ameliorate learning and memory deficits in this FXS model. However, the serotonin receptor involved in this mechanism needed to be tested. One candidate is serotonin receptor 2A (5HT_2A_R)^57^, which is strongly implicated in learning/memory function^21^. To test 5HT_2A_R requirements, we used a UAS-*5HT*_*2A*_*R* RNAi^34,43^ driven in the *dfmr1* null mutant using the same three Gal4 drivers employed above. All *5-HT*_*2A*_*R* RNAi genotypes were tested alongside Gal4 drivers alone (*UH1*-Gal4/*w*^*1118*^, *elav*-Gal4/*w*^*1118*^, *repo*-Gal4/*w*^*1118*^) as well as the UAS transgene alone (UAS-*5-HT*_*2A*_*R* RNAi/* w*^*1118*^), which all performed at the *w*^*1118*^ level (Fig. 1C; Fig. S3). Compared to *w*^*1118*^ background control, *dfmr1* nulls again have significantly lower learning (*p* = 7.460 × 10^–5^) and memory (*p* = 8.599 × 10^–5^) behavior performance (Fig. 5A,B). In learning, ubiquitous *5HT*_*2A*_*R* knockdown with *UH1*-Gal4 only slightly, albeit significantly, increases the *dfmr1* null mutant impairment (*p* = 0.01548) which, together with the strong *5HT*_*2A*_*R* RNAi impact alone in the control background (*p* = 2.588 × 10^–4^; Fig. 1C), suggests a large contribution of *5HT*_*2A*_*R* loss to the FXS model deficit (Fig. 5A). Global *UH1*-Gal4 driven *5HT*_*2A*_*R* RNAi in *dfmr1* nulls remains significantly impaired relative to *w*^*1118*^ (*p* = 4.6 × 10^–10^), suggesting a critical requirement in immediate learning. The neuronal *elav*-Gal4 outcome is not significantly different from *UH1*-Gal4 (*p* = 0.9093), but glial *repo*-Gal4 is significantly different (*p* = 9.559 × 10^–3^), showing the *5HT*_*2A*_*R* requirement is solely in neurons (Fig. 5A). For memory, ubiquitous *5HT*_*2A*_*R* RNAi causes no difference from *dfmr1* null mutants alone (*p* = 0.8586), remaining significantly impaired versus *w*^*1118*^ controls (*p* = 7.094 × 10^–7^; Fig. 5B). There are no significant differences between or within the three Gal4 drivers. These findings suggest neuronal 5HT_2A_R enables learning in concert with *dfmr1* function.

To test 5HT_2A_R expression in the MB learning circuit, dual anti-5HT_2A_R and -Trio co-labeling was done in both untrained and T-maze trained conditions (Fig. 5C). Genetic background controls (*w*^*1118*^) exhibit high 5HT_2A_R puncta within and around the MB lobes compared to strongly reduced 5HT_2A_R puncta in the FXS disease model (Fig. 5C, left). Knockdown of *5HT*_*2A*_*R* with all Gal4 drivers nearly abolishes detectable 5HT_2A_R labeling, suggesting interconnected 5HT_2A_R maintenance in the neurons and glia (Fig. 5C, right). T-maze aversive conditioning training does not detectably alter MB 5HT_2A_R levels in any of the five genotypes (Fig. 5C, bottom row). Quantification of anti-5HT_2A_R fluorescence intensity within the MB lobes reveals a significant decrease in the *dfmr1* null mutants compared to the *w*^*1118*^ background controls (*p* = 7.858 × 10^–3^; Fig. 5D), suggesting the FXS model has lower 5HT_2A_R levels in the learning circuit. Control genotypes (*UH1*-Gal4/*w*^*1118*^, *elav*-Gal4/*w*^*1118*^, *repo*-Gal4/*w*^*1118*^, and UAS-*5-HT*_*2A*_*R* RNAi/*w*^*1118*^) show 5-HT_2A_R levels comparable to *w*^*1118*^ alone, confirming a reduction specific to *dfmr1* loss. In the untrained condition, ubiquitous *5HT*_*2A*_*R* knockdown in the *dfmr1* null mutants causes significantly reduced fluorescence intensity compared to both *w*^*1118*^ controls (*p* = 7.621 × 10^–9^) and *dfmr1* nulls (*p* = 5.732 × 10^–3^; Fig. 5D, right). No significant differences occur between the three Gal4 drivers. Following T-maze training, *dfmr1* mutants continue to exhibit reduced 5HT_2A_R level compared to *w*^*1118*^ controls (*p* = 9.630 × 10^–3^), consistent with basal conditions (Fig. 5E). Likewise, 5HT_2A_R levels remain significantly reduced with *UH1*-driven *5HT*_*2A*_*R* RNAi in *dfmr1* nulls compared to *dfmr1* mutants alone (*p* = 2.454 × 10^–3^) and *w*^*1118*^ controls (*p* = 5.083 × 10^–9^; Fig. 5E). No significant differences are detected among the Gal4 drivers. These findings show a loss of 5HT_2A_ receptors in *dfmr1* mutants, and suggest *5HT*_*2A*_*R* overexpression should rectify *Drosophila* FXS disease model phenotypes.

### *5HT*_*2A*_*R* overexpression restores learning and memory in the FXS disease model

To test whether elevating serotonin receptor 2A (5HT_2A_R) signaling can restore learning and memory in *dfmr1* nulls, we overexpressed UAS-*5HT*_*2A*_*R*^43^. Our hypothesis is that 5HT_2A_R loss in the FXS disease model results in impaired learning and memory, which should be corrected by potentiating 5-HT signaling either by elevating 5-HT levels (Figs. 2, 3, and 4) or increasing 5HT_2A_R expression. All 5-HT_2A_R overexpression genotypes were tested alongside Gal4 driver alone (*UH1*-Gal4/*w*^*1118*^, *elav*-Gal4/*w*^*1118*^, *repo*-Gal4/*w*^*1118*^) and UAS-transgene (UAS-*5-HT*_*2A*_*R*/* w*^*1118*^) controls, which all performed at *w*^*1118*^ levels. Compared to genetic background controls (*w*^*1118*^), *dfmr1* nulls once again show a very significantly impaired learning (*p* = 4.112 × 10^–7^) and memory (*p* = 2.133 × 10^–5^) performance (Fig. 6A,B). For learning, ubiquitous UAS-*5HT*_*2A*_*R* overexpression with the *UH1*-Gal4 driver in the *dfmr1* null significantly restores memory performance relative to *dfmr1* alone (*p* = 2.404 × 10^–5^), to a level not significantly different from the *w*^*1118*^ controls (*p* = 0.8517), indicating a complete behavioral correction (Fig. 6A). *UH1*-driven *5HT*_*2A*_*R* overexpression results in significantly better performance than the *repo*-driven receptor (*p* = 8.799 × 10^–3^), suggesting a selective 5HT_2A_R role in neurons. Consistently, there is no significant difference between *UH1-* and *elav-*Gal4 *5HT*_*2A*_*R* overexpression (*p* = 0.3961), indicating neuronal 5HT_2A_R function (Fig. 6A). For memory performance, the *UH1*-driven *5HT*_*2A*_*R* overexpression significantly improves behavioral outcomes compared to *dfmr1* null mutant alone (*p* = 2.762 × 10^–4^), with the restored memory performance not significantly different from the *w*^*1118*^ controls (*p* = 0.9604), indicating a complete behavioral rectification (Fig. 6B). There is no significant difference between the three Gal4 drivers. Transgenic controls (*UH1*-Gal4/*w*^*1118*^, *elav*-Gal4/*w*^*1118*^, *repo*-Gal4/*w*^*1118*^, and UAS-*5HT*_*2A*_*R*/*w*^*1118*^) show no significant behavioral differences from *w*^*1118*^, confirming rescue is specific to *5-HT*_*2A*_*R* overexpression (Fig. S3). These findings indicate *5-HT*_*2A*_*R* overexpression can strongly restore both learning and memory performance in the *Drosophila* FXS disease model.

To test 5HT_2A_R levels in the MB circuit, brains from all five genotypes were double labeled for anti-Trio and anti-5HT_2A_R in both untrained and trained conditions (Fig. 6C). For best image clarity, only the MB outline derived from anti-Trio is displayed. Genetic background controls (*w*^*1118*^) show high 5HT_2A_R puncta compared to many fewer 5HT_2A_R puncta in the FXS disease model (Fig. 6C, left). *5HT*_*2A*_*R* overexpression with all three Gal4 drivers increase the 5HT_2A_R levels, again suggesting interconnected 5HT_2A_R expression in MB neurons and glia (Fig. 6C, right). T-maze aversive conditioning training does not detectably alter 5HT_2A_R levels in the MB circuit of any of the tested genotypes (Fig. 6C, bottom row). Quantification of anti-5HT_2A_R fluorescence intensity within the MB shows a very significant loss in *dfmr1* null mutants compared to the *w*^*1118*^ background controls (*p* = 3.495 × 10^–3^; Fig. 6D), again indicating the FXS disease model has reduced 5HT_2A_R levels in the MB circuit. Control genotypes (*UH1*-Gal4/*w*^*1118*^, *elav*-Gal4/*w*^*1118*^, *repo*-Gal4/*w*^*1118*^, and UAS-*5HT*_*2A*_*R*/*w*^*1118*^) display 5-HT_2A_R levels comparable to *w*^*1118*^, indicating the observed increase is specific to 5-HT_2A_R overexpression. In the basal, untrained condition, *UH1*-driven *5HT*_*2A*_*R* overexpression in the *dfmr1* null mutants very significantly elevates MB 5HT_2A_R levels compared to *dfmr1* alone (*p* = 4.722 × 10^–8^) and more weakly compared to the *w*^*1118*^ background controls (*p* = 2.067 × 10^–2^), confirming successful overexpression (Fig. 6D). There are no significant differences between the Gal4 drivers. Following T-maze training, the same pattern is present with significantly lower MB 5HT_2A_R levels in *dfmr1* mutants compared to matched controls (*p* = 1.423 × 10^–2^) and a very significant increase with *5HT*_*2A*_*R* overexpression in *dfmr1* null mutants, strongly compared to *dfmr1* alone (*p* = 3.127 × 10^–8^) and more weakly compared to *w*^*1118*^ controls (*p* = 6.309 × 10^–3^; Fig. 6E). Taken together, these findings indicate that loss of 5HT_2A_R signaling causes learning and memory deficits in *dfmr1* mutants and that 5HT_2A_R overexpression can correct these impairments in the *Drosophila* FXS disease model.

## Discussion

Fragile X syndrome (FXS) results from epigenetic loss of the Fragile X Messenger Ribonucleoprotein (FMRP), an mRNA-binding protein tightly regulating the translation of proteins critical for synaptic plasticity^58,59^. Like FXS patients, mouse and *Drosophila* FXS models exhibit deficient learning/memory^60,61^, behaviors known to be modulated by serotonin^20,62^. Using classical olfactory aversive conditioning^44^, we find loss of FMRP and 5-HT_2A_R similarly impairs learning and memory (Fig. 1). Elevating serotonin via overexpression of the biosynthetic enzyme Trhn (Fig. 2) or SERT transporter knockdown (Fig. 3) restores learning and memory performance to control levels within detectable limits in the *dfmr1* null mutants. These complementary approaches are equally efficacious in correcting both of the behavioral outcomes, although Trhn overexpression drives much higher serotonin levels in the Mushroom Body (MB) learning/memory circuit (Fig. 4). The *Drosophila* FXS model shows reduced 5-HT_2A_R levels, and *5-HT*_*2A*_*R* knockdown only weakly exacerbates the *dfmr1* null mutant learning impairment, without any worsening of the memory deficit (Fig. 5). Consistently, *5-HT*_*2A*_*R* overexpression within our FXS model restores normal learning and memory in *dfmr1* null mutants (Fig. 6). Thus, the *Drosophila* FXS disease model has strongly impaired 5-HT_2A_R signaling, and elevating either 5-HT or 5-HT_2A_R levels effectively restores *Drosophila* FXS disease learning and memory performance. Note all conclusions are based on associative olfactory conditioning and focus specifically on the Mushroom Body circuit, and therefore may not reflect broader FXS processes.

Null *dfmr1* mutants exhibit significant deficits in classical olfactory conditioning learning and long-term memory (Fig. 1A–C). These results agree with previous studies in the *Drosophila* FXS model^7,8^, the mouse FXS model^62,63^, and in human FXS patients^64,65^. Serotonergic neuromodulation is a conserved cognitive mechanism in all three cases^17,22,66^. Among serotonin receptors, 5-HT_2A_R signaling is recognized as an important modulator of learning acquisition and memory consolidation in mammals^21^. However, 5-HT_2A_R roles in learning and memory had not been previously tested in *Drosophila*. Here, we find that *5-HT*_*2A*_*R* knockdown impairs both behaviors (Fig. 1A–C). Elevating 5-HT levels by itself does not enhance performance outcomes, although serotonergic signaling is known to regulate *Drosophila* learning and memory^22^. In the *Drosophila* FXS model, the underlying MB circuit (Fig. 1D–E) shows defects in experience-dependent Kenyon cell structure and function^5,11,67^ consistent with serotonergic impairments. Fully-mapped brain 3-dimensional neural circuit reconstruction^68^ reveals MB-innervating serotonergic neurons (Fig. 1E) that are well positioned to control learning and memory behavior in this system. Note this study focuses on a specific brain circuit and behavior, so cannot capture the full complexity of the FXS disease condition, and that further work is needed to clarify precise molecular, cellular, and circuit serotonergic mechanisms underlying broader FXS effects.

Elevating serotonin levels—either by overexpressing the rate-limiting serotonin synthesis enzyme Trhn (Fig. 2) or by knockdown of the serotonin reuptake transporter SERT (Fig. 3)—restores FXS learning and memory performance to the control levels. Global (*UH1*-Gal4), neural (*elav*-Gal4), and glial (*repo*-Gal4) drivers produce comparable correction, which is surprising, but consistent with neurons and glia both using Trhn and SERT to regulate serotonin levels^30,34^. Similar serotonergic-based effects occur in the mouse FXS model, although direct parallels must be interpreted cautiously, as serotonin receptor distributions and circuit functions differ between models. Specifically, mice with psilocybin can ameliorate cognitive deficits in *Fmr1* knockout mice^62^. SSRI treatments (e.g. sertraline) can likewise improve cognitive function in FXS children^69^, supporting a conserved role for 5-HT elevation correcting FXS-related impairments. In the *Drosophila* FXS model, 5-HT levels are not altered in the MB learning/memory circuit, but Trhn overexpression and SERT knockdown elevate 5-HT in *dfmr1* nulls (Figs. 2, 3). Basal 5-HT levels are similar in the controls and *dfmr1* nulls, but increasing 5-HT levels is sufficient to activate the reduced number of 5-HT_2A_Rs present in the FXS model. Thus, the defect is not a loss of serotonin itself, but rather reduced 5-HT_2A_R abundance, and the behavioral deficits appear to arise from insufficient receptor availability rather than loss of ligand. Parallel findings in neural and glial drivers agree with Trhn and SERT in both cell types controlling serotonin levels^30^. Additional *Drosophila* glial mechanisms (e.g. arylalkylamine N-acetyltransferase 1) can also modulate 5-HT levels^70^. Mammalian glia also control serotonin homeostasis and signaling^71,72^. Our results indicate that raising 5-HT to levels very substantially above baseline via neuronal or glial mechanisms restores learning/memory behavior in the *Drosophila* FXS model, although whether this finding is generalizable remains to be determined.

Comparing these two strategies for increasing serotonin availability shows both restore learning and memory in the FXS model (Fig. 4A,B). This behavioral correction consistency suggests both methods converge on a shared outcome of higher 5-HT ligand levels, rather than targeting separable steps within the serotonergic regulatory pathway^73^. Our results argue against SERT being a principal site of FMRP action, for example, since reducing SERT does not yield different behavioral outcomes compared to Trhn overexpression. Blocking SERT using SSRIs is reportedly effective in cognitive preclinical trials^74,75^. Similarly, increasing serotonin via dietary tryptophan supplementation reportedly improves memory in cognitively impaired patients^76,77^. Elevating 5-HT has been suggested to compensate for 5-HT receptor loss to improve cognitive abilities^77^. Trhn overexpression is more effective than SERT knockdown in elevating serotonin levels in the MB learning/memory circuit (Fig. 4C,D). The absence of a training-induced 5-HT change indicates behavioral phenotypes do not arise from acute serotonin modulation by conditioning, but rather reflect differences in baseline serotonergic tone that determine the system capacity to support learning and memory (Fig. 4C,D). Although olfactory conditioning T-maze training has no discernible effect on 5-HT levels, neuronal and glial drivers are equally effective, with a trend toward an additive effect occurring with the ubiquitous driver. This result is surprising, but consistent with neurons and glia both using Trhn and SERT for serotonin signaling^30,34^. Importantly, behavioral changes from elevating 5-HT are observed only in *dfmr1* mutants in our studies, indicating that these manipulations act to correct a deficit specific to the FXS model rather than producing a general enhancement of performance. These results prompted us to focus on the downstream 5-HT receptor mechanism in our FXS disease model.

*5-HT*_*2A*_*R* knockdown impairs learning and memory (Fig. 1), but in *dfmr1* nulls has a minimal additional effect on learning and no significant additive effect on memory (Fig. 5A,B). The 5-HT_2A_R role is within neurons, with no function in glia. These results indicate FXS model behavioral impairments are mediated primarily through lost neuronal 5-HT_2A_R signaling. Previous *Drosophila* studies have shown 5-HT_2A_R signaling regulates locomotion^78^, metabolic activity^79^, and synapse pruning^30,34^, but no prior work assessed roles in learning or memory. However, mouse 5-HT_2A_Rs are involved in synaptic plasticity in mechanisms underlying cognition^80–82^. Human 5-HT_2A_Rs regulate cortical function linked to cognitive impairment across multiple psychiatric conditions^21,83^. In the *Drosophila* FXS model, there is a significant 5-HT_2A_R loss in the MB circuit (Fig. 5C–E). Just like for the 5-HT ligand, T-maze olfactory conditioning training has no discernible effect on the 5-HT_2A_R levels. Similarly, lack of training-dependent modulation of 5-HT_2A_Rs supports a model of fixed baseline 5-HT_2A_R level, rather than dynamic regulation during conditioning, determining the efficiency of serotonergic signaling required for associative learning. Both neuronal and glial drivers similarly knockdown 5-HT_2A_Rs, consistent with receptors being present in both cell types^30,34^. This suggests a regulatory maintenance mechanism within the MB circuit^84^. Previous studies have shown 5-HT_2A_Rs expressed in the MB circuit^53^, but this work now connects 5-HT_2A_R signaling to learning/memory or FXS model behavioral dysfunction.

*5-HT*_*2A*_*R* overexpression in *dfmr1* nulls is sufficient to restore learning and memory in our FXS disease model (Fig. 6A,B), which strongly reinforces the conclusion that serotonergic 5-HT_2A_R signaling corrects behavioral impairments. 5-HT_2A_Rs act primarily in neurons, but glial signaling also contributes, again suggesting balanced circuit-level modulation of receptor function^30^. We previously discovered glial *5-HT*_*2A*_*R* overexpression allows experience-dependent synaptic connectivity remodeling^34^, but there are no other studies of 5-HT_2A_R overexpression in *Drosophila*. However, *5-HT*_*2A*_*R* overexpression in *5-HT*_*2A*_*R* knockout mice medial dorsal thalamus restores associative memory, demonstrating that enhancing 5-HT_2A_R function can directly rescue cognitive deficits in mammals^85^. Pharmacological activation of 5-HT_2A_Rs using agonists including 2,5-dimethoxy-4-iodoamphetamine (DOI), psilocybin, and LSD has been shown to promote neuroplasticity and ‘cognitive flexibility’, supporting potential applications for treating cognitive impairments^86–88^. In the *Drosophila* MB learning/memory circuit, 5-HT_2A_R levels are independently confirmed as reduced in the FXS disease model and also elevated by transgenic overexpression in the *dfmr1* null mutants (Fig. 6C–E). 5-HT_2A_R levels are elevated in all three of the transgenic drivers, with no significant differences comparing the untrained and associatively trained animals. These findings are consistent with a 5-HT_2A_ receptor-limited mechanism, in which the reduced 5-HT_2A_R level in *dfmr1* null mutants constrains serotonergic signaling within the learning/memory circuit, with highly elevated 5-HT levels overcoming the receptor deficit. These abnormally high 5-HT levels could conceivably induce neurotrophic or plasticity-promoting factors to artificially drive correction of learning and memory impairments in our FXS model.

In *Fmr1* knockout mice, abnormal 5-HT_2A_ receptor function occurs in cortical circuits^36^, and pharmacological 5-HT_2A_R blockade ameliorates learning deficits^89^. However, serotonergic receptor distribution, downstream signaling, and circuit architectures differ substantially between mice and *Drosophila*. Thus, while both FXS models reveal 5-HT_2A_R signaling disrupted by FMRP loss, the consequences and directionality of disruption depend on species-specific circuit contexts. Thus, our results define a 5-HT_2A_R signaling role in the *Drosophila* FXS model, and do not contradict the distinct 5-HT receptor phenotypes observed in mice. Besides 5-HT_2A_R, both 5-HT_1A_ and 5-HT_7_ receptors are expressed in the *Drosophila* brain^90^. 5-HT_1A_R activation has been shown to rescue mitochondrial malfunction and motor impairments in the *Drosophila* FXS model^91^, indicating that multiple 5-HT receptors are affected. Importantly, 5-HT_7_R signaling plays a role in olfactory associative learning, with MB 5-HT_7_Rs mediating cAMP-dependent learning^92^. Our work identifies 5-HT_2A_R loss in the *Drosophila* FXS model MB circuit, but other 5-HT receptors could participate in the 5-HT_2A_R-mediated effect on learning and memory. Since 5-HT ligand levels are unchanged in *dfmr1* mutants, and because behavioral restoration aligns precisely with manipulations that compensate for reduced 5-HT_2A_R signaling, the simplest interpretation is that learning/memory impairments in this aversive conditioning task arise from insufficient 5-HT_2A_R-mediated signaling. Thus, although 5-HT_1A_ and 5-HT_7_ receptors likely contribute to serotonergic functions in disease contexts, the combined molecular and behavioral evidence presented here identifies 5-HT_2A_R as the key receptor mediating FXS-related deficits in MB-dependent learning and memory.

In conclusion, this investigation demonstrates that elevated serotonergic signaling corrects learning and memory impairments in the *Drosophila* Fragile X syndrome model. By transgenically increasing the 5-HT ligand levels (*Trhn* overexpression synthesis, *SERT* knockdown blocking uptake), serotonin is elevated to overcome a 5-HT_2A_R limitation in the MB learning/memory circuit with learning and memory behavior restored in *dfmr1* nulls.

These findings support a baseline-tone mechanism, with learning and memory capacity determined by sufficient 5-HT_2A_R levels, not dynamic signaling effects, acting to limit the signaling capacity rather than direct circuit plasticity in response to training. In future work, 5-HT_2A_Rs could be selectively manipulated in defined serotonergic neurons (e.g. DPMs), using transgenic drivers to target the specific cells determining the serotonergic receptor-mediated signaling capacity. Importantly, 5-HT_2A_Rs are downregulated in our FXS model, *5-HT*_*2A*_*R* knockdown alone phenocopies *dfmr1* null mutant behavioral impairments, and *5-HT*_*2A*_*R* knockdown in the FXS disease model only slightly exacerbates learning and memory deficits. These discoveries indicate that 5-HT_2A_R signaling dysfunction appears causative in FXS disease model behavioral impairments. In future work, systemic use of SSRIs and 5-HT_2A_R agonists could be attempted to assess whether the pharmacological activation of serotonergic signaling is sufficient to ameliorate learning and/or memory deficits in *dfmr1* mutants. Finally, inducing *5-HT*_*2A*_*R* overexpression in our FXS disease model elevates receptor levels in the MB learning/memory circuit and corrects behavioral impairments in *dfmr1* mutants. These findings suggest serotonergic 5-HT_2A_R signaling may be a useful means for FXS intervention strategies, pending future investigation in mammalian systems, and could provide a basis for future FXS therapeutic treatments.

## Supplementary Information

Supplementary Information 1.

Supplementary Information 2.

Supplementary Information 3.

Supplementary Information 4.

Supplementary Information 5.

## Data Availability

All original data analyzed during this study are publicly available within the Harvard Dataverse under the “Kendal Broadie Dataverse” heading. [https://dataverse.harvard.edu/dataverse/kendalbroadie].
