# Supplementary material for: Elevated serotonin receptor 2A signaling restores learning and memory in a Fragile X syndrome model
Source: Sci Rep. 2026 Jan 7;16:4450. doi: 10.1038/s41598-025-34492-4 (PMC12864886; doi:10.1038/s41598-025-34492-4)
Supplement: Supplementary file 1 — Supplementary Information 1. [file 41598_2025_34492_MOESM1_ESM.docx]

**Supplementary Figure Legends**

**Table S1:**  **Sample sizes and statistical analysis results for all quantified datasets**

For each genotype (*w^1118^* control, *dfmr1* null, and *dfmr1* with *UH1-*, *elav-*, or *repo-*Gal4 driven *Trhn* overexpression, SERT RNAi, *5HT_2A_R* RNAi, and *5HT_2A_R* overexpression) and treatment condition, the number of trials/samples (n) and mean ± SEM values are provided here. All values correspond directly to the behavioral and fluorescence intensity measurements shown in the figures, as indicated in the table.

**Figure S1: Anti-Trio immunolabeling delineating the Mushroom Body (MB) lobes**

For each genotype and condition displayed in Figure 2C (*w^1118^* control, *dfmr1* null, and *dfmr1* with *UH1-*, *elav-*, or *repo-*Gal4 driven *Trhn* overexpression), the anti-Trio labeling used to reveal the MB lobe outlines employed for the 5-HT quantification. All images represent the same panels as Figure 2C marking the MB (green outlines in Figure 2C), collected using identical confocal microscope acquisition settings.

**Figure S2: Mushroom Body circuit 5-HT levels do not differ in control genotypes**

Quantification of MB anti-5-HT fluorescence intensities across all control genotypes for *Trhn* overexpression and *SERT* RNAi conditions used in Figures 2-4. For each genotype (*w^1118^*, *UH1*-Gal4/*w^1118^*, *elav*-Gal4/*w^1118^*, *repo*-Gal4/*w^1118^*, UAS-*Trhn*/*w^1118^*, and UAS-*SERT* RNAi/*w^1118^*), independent brain (n=5-11/condition) data sets show mean ± SEM, and one-way ANOVA with Tukey’s multiple comparisons tests. No significant differences occur in any comparison (F(5,33) = 0.3899). Significance indicated as *p*>0.05 (not significant, ns).

**Figure S3: Learning and memory indices do not differ between control genotypes**

Performance indices of learning (**A**) and memory (**B**) for the five control genotypes in the *5HT_2A_R* RNAi and overexpression conditions from Figures 5-6. Genotypes include *w^1118^*, *UH1*-Gal4/*w^1118^*, *elav*-Gal4/*w^1118^*, *repo*-Gal4/*w^1118^*, UAS-*5HT_2A_R* RNAi/*w^1118^*, and UAS-*5HT_2A_R*/*w^1118^*. Data show individual trials (n=8-16/condition), mean ± SEM, and one-way ANOVA with Tukey’s multiple comparisons tests. No significant differences occur between any genotypes for learning (F(5,50) = 1.381) or memory (F(5,50) = 0.8544) between any of the genotypes. Significance is indicated as *p*>0.05 (not significant, ns).
