# Supplementary figures and images for: Elevated serotonin receptor 2A signaling restores learning and memory in a Fragile X syndrome model
Source: Sci Rep. 2026 Jan 7;16:4450. doi: 10.1038/s41598-025-34492-4 (PMC12864886; doi:10.1038/s41598-025-34492-4)

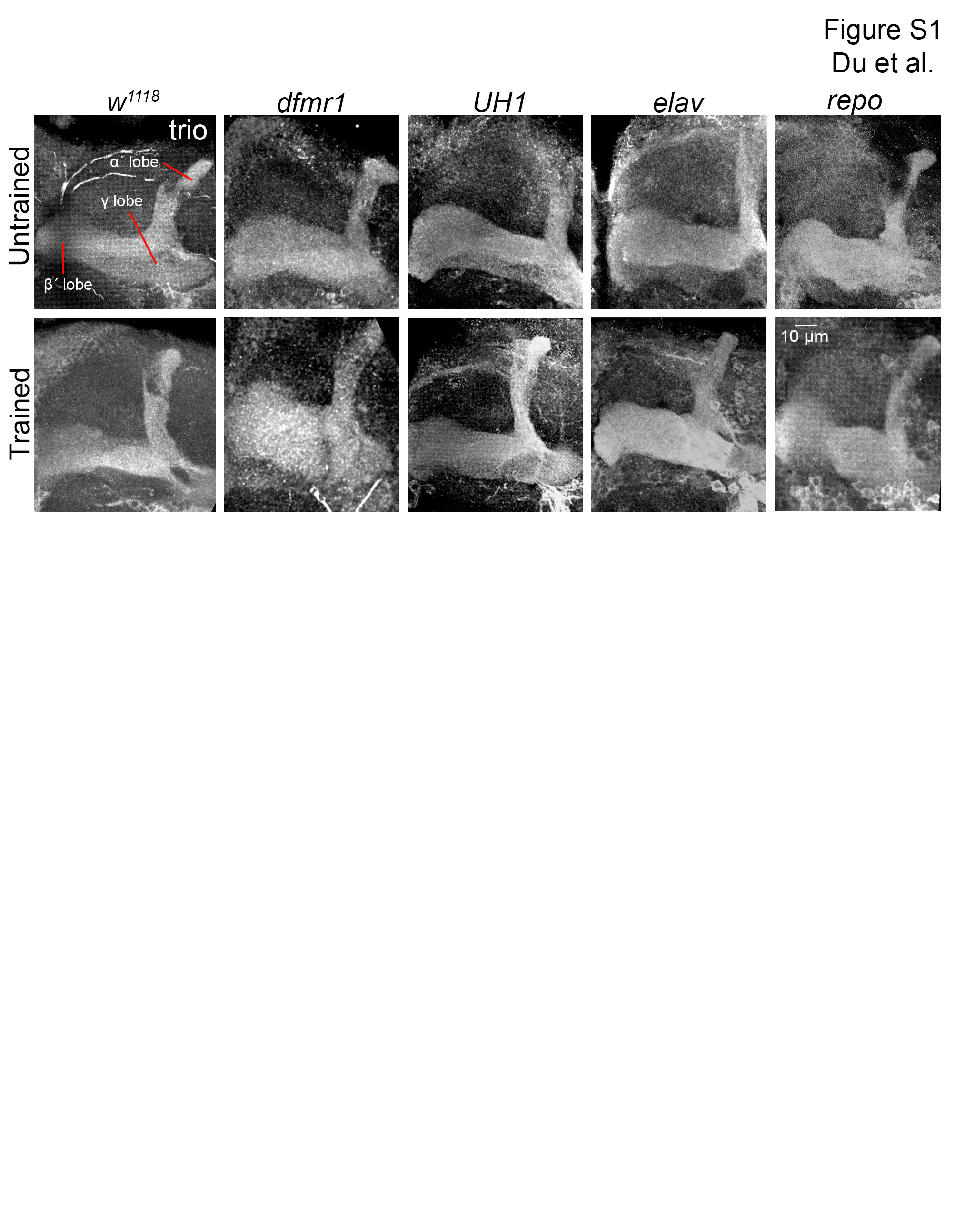

Supplement: Supplementary file 3 — Supplementary Information 3. [file 41598_2025_34492_MOESM3_ESM.tif]

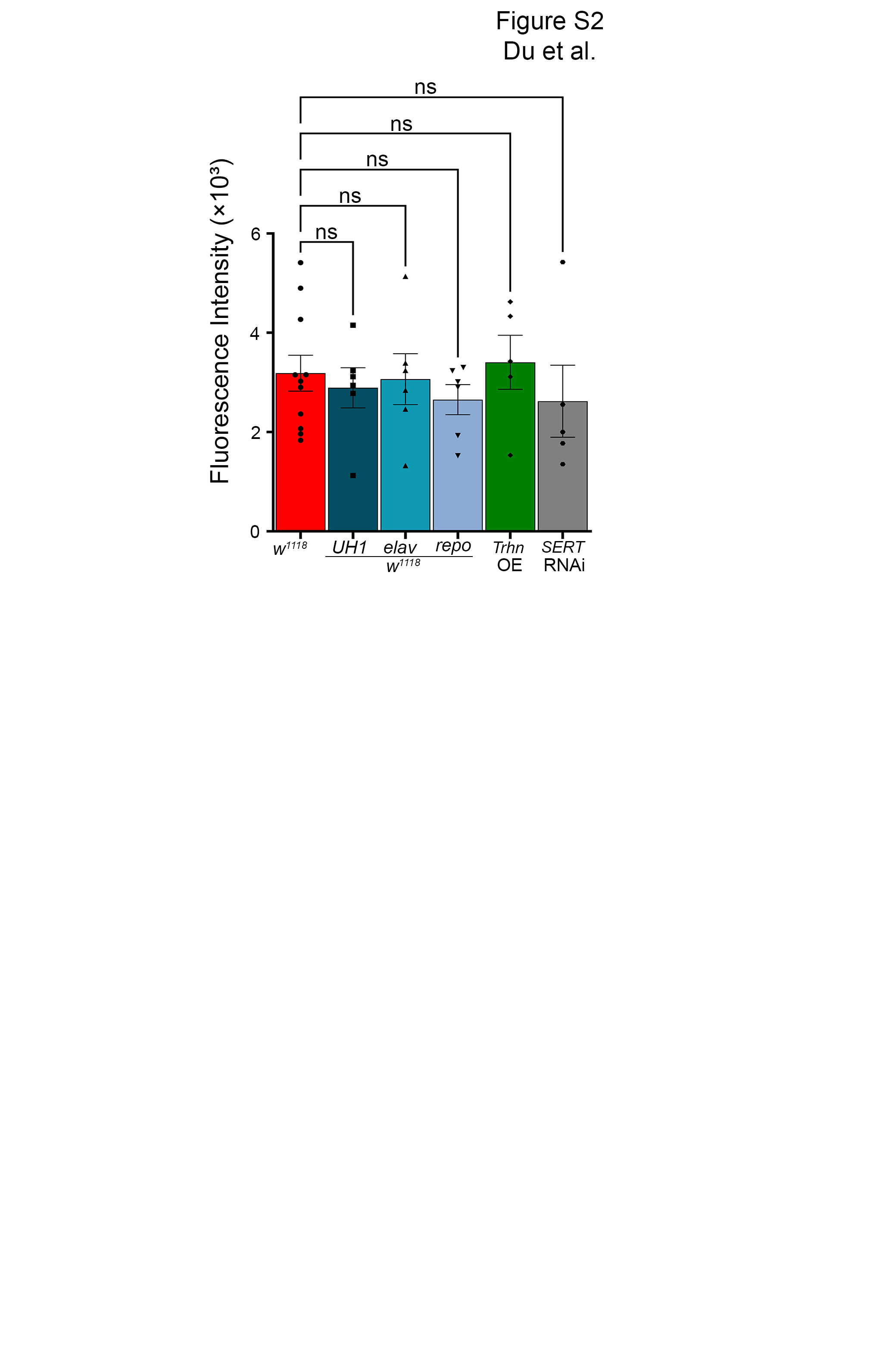

Supplement: Supplementary file 4 — Supplementary Information 4. [file 41598_2025_34492_MOESM4_ESM.tif]

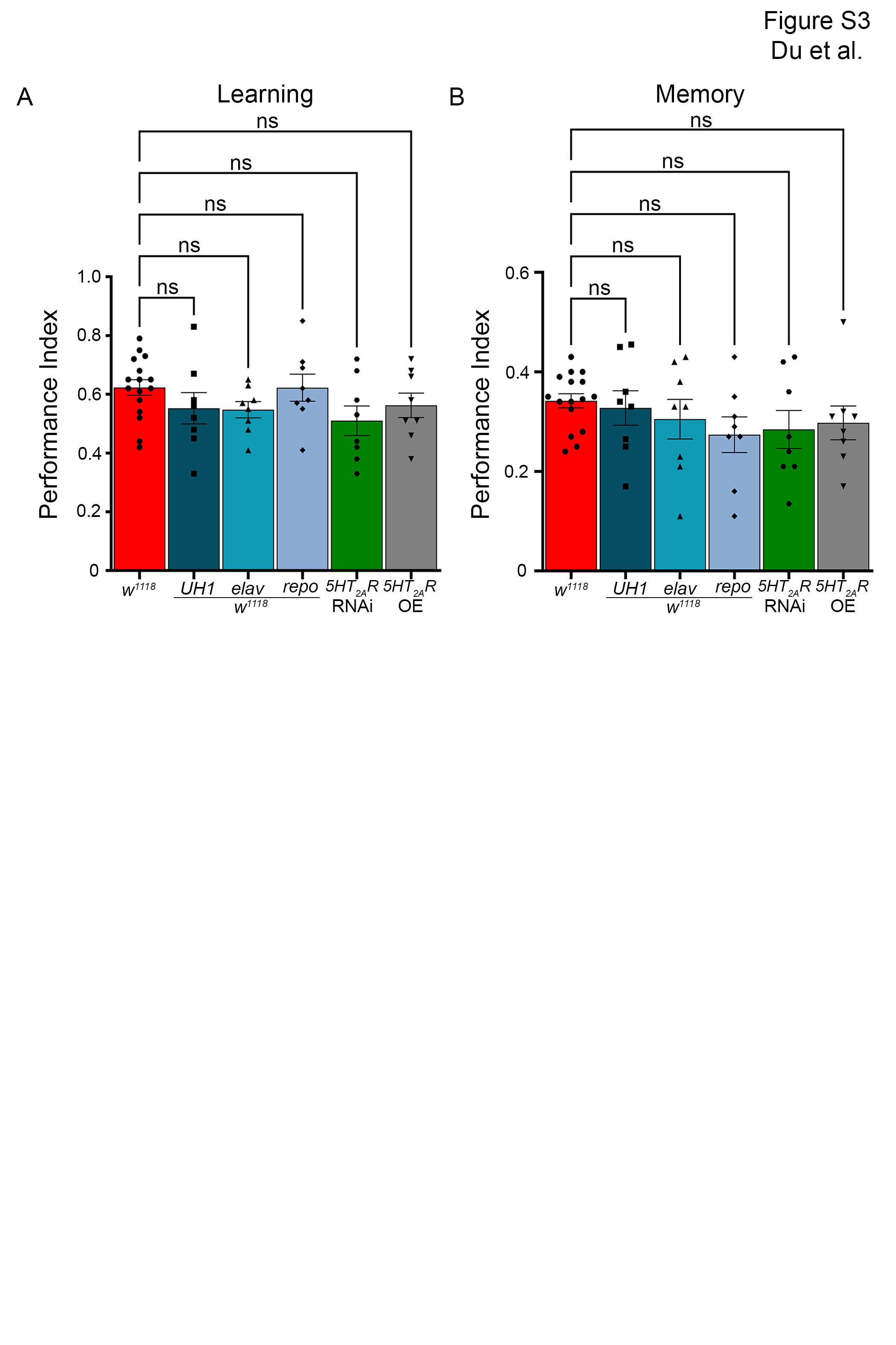

Supplement: Supplementary file 5 — Supplementary Information 5. [file 41598_2025_34492_MOESM5_ESM.tif]
